# Expression profile of HERVs and inflammatory mediators detected in nasal mucosa as a predictive biomarker of COVID-19 severity

**DOI:** 10.3389/fmicb.2023.1155624

**Published:** 2023-05-22

**Authors:** Vita Petrone, Marialaura Fanelli, Martina Giudice, Nicola Toschi, Allegra Conti, Christian Maracchioni, Marco Iannetta, Claudia Resta, Chiara Cipriani, Martino Tony Miele, Francesca Amati, Massimo Andreoni, Loredana Sarmati, Paola Rogliani, Giuseppe Novelli, Enrico Garaci, Guido Rasi, Paola Sinibaldi-Vallebona, Antonella Minutolo, Claudia Matteucci, Emanuela Balestrieri, Sandro Grelli

**Affiliations:** ^1^Department of Experimental Medicine, University of Rome Tor Vergata, Rome, Italy; ^2^Department of Biomedicine and Prevention, University of Rome Tor Vergata, Rome, Italy; ^3^Martinos Center for Biomedical Imaging and Harvard Medical School, Boston, MA, United States; ^4^Department of Systems Medicine, University of Rome Tor Vergata, Rome, Italy; ^5^Respiratory Medicine Unit, Policlinic of Tor Vergata, Rome, Italy; ^6^Neuromed IRCCS Institute, Pozzilli, IS, Italy; ^7^University of Nevada, Department of Pharmacology, Reno, NV, United States; ^8^IRCCS San Raffaele Pisana, Rome, Italy; ^9^National Research Council, Institute of Translational Pharmacology, Rome, Italy; ^10^Virology Unit, Policlinic of Tor Vergata, Rome, Italy

**Keywords:** human endogenous retroviruses, HERV, biomarker, respiratory outcome, inflammation, COVID-19

## Abstract

**Introduction:**

Our research group and others demonstrated the implication of the human endogenous retroviruses (HERVs) in SARS-CoV-2 infection and their association with disease progression, suggesting HERVs as contributing factors in COVID-19 immunopathology. To identify early predictive biomarkers of the COVID-19 severity, we analyzed the expression of HERVs and inflammatory mediators in SARS-CoV-2-positive and -negative nasopharyngeal/oropharyngeal swabs with respect to biochemical parameters and clinical outcome.

**Methods:**

Residuals of swab samples (20 SARS-CoV-2-negative and 43 SARS-CoV-2-positive) were collected during the first wave of the pandemic and expression levels of HERVs and inflammatory mediators were analyzed by qRT-Real time PCR.

**Results:**

The results obtained show that infection with SARS-CoV-2 resulted in a general increase in the expression of HERVs and mediators of the immune response. In particular, SARS-CoV-2 infection is associated with increased expression of HERV-K and HERV-W, IL-1β, IL-6, IL-17, TNF-α, MCP-1, INF-γ, TLR-3, and TLR-7, while lower levels of IL-10, IFN-α, IFN-β, and TLR-4 were found in individuals who underwent hospitalization. Moreover, higher expression of HERV-W, IL-1β, IL-6, IFN-α, and IFN-β reflected the respiratory outcome of patients during hospitalization. Interestingly, a machine learning model was able to classify hospitalized *vs* not hospitalized patients with good accuracy based on the expression levels of HERV-K, HERV-W, IL-6, TNF-a, TLR-3, TLR-7, and the N gene of SARS-CoV-2. These latest biomarkers also correlated with parameters of coagulation and inflammation.

**Discussion:**

Overall, the present results suggest HERVs as contributing elements in COVID-19 and early genomic biomarkers to predict COVID-19 severity and disease outcome.

## 1. Introduction

The global COVID-19 pandemic, caused by the novel severe acute respiratory syndrome coronavirus 2 (SARS-CoV-2) (Wu et al., 2020), has strained the healthcare systems all around the world, emphasizing the urgent challenge of defining the pathogenesis of the disease and identifying biomarkers predictive of the clinical evolution.

Like other coronaviruses, SARS-CoV-2 infection primarily targets the respiratory tract (Chu et al., 2004; Channappanavar and Perlman, 2017) by binding to angiotensin-converting enzyme 2 (ACE2). Initially, SARS-CoV-2 shows high viral replication in upper airway epithelial cells with initial symptoms, affecting that district (Chu et al., 2004). The first host response to SARS-CoV-2 is elicited at the mucosal level, where the immune microenvironment is represented by the nasopharynx-associated lymphoid tissue system (Gallo et al., 2021), which consists of T cells, B cells, dendritic cells, macrophages, and microfold cells (Krege et al., 2009; Lee et al., 2015). Thus, at the level of epithelial and immune cells, SARS-CoV-2 infection triggers an early production of type I IFNs and inflammatory cytokines (IL-6 and IL-10) (Villena and Kitazawa, 2020; Ziegler et al., 2020), resulting in dysfunction in the immune response and enhancement in the production of multiple cytokines and chemokines, which in turn elicit significant differences between favorable and unfavorable clinical evolutions (Chu et al., 2004; Channappanavar and Perlman, 2017). In particular, the altered cytolytic activity of lymphocytes results in the inability of NK cells and CD8 T cells to lyse infected cells. The prolonged and exacerbated interaction between innate and adaptive immune cells leads to the unregulated secretion of many pro-inflammatory cytokines, including TNF, interferon-γ, IL-1, IL-6, IL-18, and IL-33, causing a cytokine storm (Mehta et al., 2020) and, in some patients, acute respiratory distress syndrome (ARDS) (Soy et al., 2020). Moreover, peculiar expression profiles of SARS-CoV-2 associated host invasion genes in nasopharyngeal and oropharyngeal swabs of COVID-19 patients have been described as good discriminator of clinical outcome in COVID-19 patients with complementary role in the virus entry and in disease progression (Amati et al., 2020). Recently our research group and others have demonstrated the implication of the human endogenous retrovirus-K (HERV-K) and HERV-W in patient status and disease progression, suggesting HERVs as contributing factors in COVID-19 immunopathology (Balestrieri et al., 2021; Temerozo et al., 2022). HERVs are genetic elements, relics of ancestral germline infections by exogenous retroviruses and resulting in proviruses stably integrated into human DNA. Currently HERVs account for up to 8% of the genetic material, with extensive inter-individual variation due to copy number variations, unfixed copies, and polymorphisms (Marchi et al., 2014; Wildschutte et al., 2016; Thomas et al., 2018). During human evolution, most integrated HERVs have been silenced because of their potential to be detrimental to the host cell, and only a few were instead domesticated to serve physiological functions (Grandi and Tramontano, 2018). It has also been shown that activation of silenced HERV sequences is associated with several human diseases including cancer, neurological and neuropsychiatric diseases and infectious diseases (Ehlhardt et al., 2006; Balestrieri et al., 2018, 2019, 2021; Cipriani et al., 2018; Matteucci et al., 2018; Levet et al., 2019). Recently, activation of HERVs in SARS-CoV-2 infection and severity of COVID-19 has been demonstrated. Specifically, in the blood cells of COVID-19 patients, HERV-W envelope (ENV) mRNA and protein were found to be highly expressed and associated with disease severity and pulmonary involvement and HERV-W ENV protein expression in lymphocytes reflected the respiratory outcome during hospitalization of COVID-19 patients (Balestrieri et al., 2021). Furthermore, the expression of HERV-K was found at a high level in tracheal aspirates from COVID-19 patients under intermittent mandatory ventilation (Temerozo et al., 2022). Notable, SARS-CoV-2 infection *in vitro* induced the expression of HERV-K and HERV-W in human primary monocyte and lymphoid cells, respectively (Temerozo et al., 2022; Charvet et al., 2023), and the exposure of PBMCs to spike protein *in vitro* activated HERV-W ENV expression in association with the production of IL-6 (Balestrieri et al., 2021). To identify early predictive genomic biomarkers of the COVID-19 evolution, in the present study we investigated the expression of HERVs and inflammatory mediators and SARS-CoV-2 infection-related genes in nasopharyngeal and oropharyngeal swabs in relation to patients’ biochemical and clinical parameters.

## 2. Materials and methods

### 2.1. Sample collection

The nasopharyngeal and oropharyngeal swabs of 43 SARS-CoV-2-positive and 20 SARS-CoV-2-negative individuals were collected at Policlinico Tor Vergata, PTV (Rome, Italy), during triage at the emergency room in the period from the end of March until the beginning of May 2020, according to standard procedures. The diagnosis of SARS-CoV-2 infection was performed using the Allplex™ 2019-nCoV multiplex Real-time PCR assay (Seegene Inc., South Korea) at the Virology Unit of the PTV. The study was performed in accordance with the ethical principles of the Declaration of Helsinki and the Guidelines for Good Clinical Practice. The ethical committee of Tor Vergata University/Hospital approved this study (protocol number: COVID_SEET prot.7562/2020), and informed written consent was obtained from each individual included in the study.

### 2.2. *In vitro* stimulation with SARS-CoV-2 spike protein in the FaDu cell line

The hypopharyngeal carcinoma cell line FaDu (kindly provided by professor Ira-Ida Skvortsova, Medical University of Innsbruck Tyrolean Cancer Research Institute, Innsbruck, Austria) was grown in MEM EAGLE medium (PAN-Biotech, Aidenbach, Bavaria) supplemented with Earle’s BSS, 2 mM L-glutamine, 1 mM sodium pyruvate, NEAA, 1.5 g/L sodium bicarbonate, 100 U/mL penicillin, 0.1 mg/mL streptomycin and 10% fetal bovine serum (PAN-Biotech). Cells were maintained at 37°C in a humidified 5% CO_2_ atmosphere and passed twice weekly after detachment with trypsin (0.025%) and EDTA solution (0.02%) in PBS (Sigma). For the *in vitro* stimulation, FaDu cells were plated at 1.5 × 10^5^ in triplicate in 24-well plates and exposed to SARS-CoV-2 Spike protein active trimer at 5 nM (BIOSYSTEM Acro, Bay Area, CA) for 3 h, 8 h, and 24 h. At the end of the incubation period, the cells were detached, washed twice in PBS, and pellets were stored at −80°C until RNA extraction.

### 2.3. RNA purification from swab samples and FaDu cells

RNA from residual swab samples and FaDu cells was purified by using a GRS total RNA kit (Total RNA Kit—Blood & Cultured cells—GRiSP, Porto Portugal) according to the manufacturer’s instructions. Briefly, 50 μL of residual swab samples or FaDu cells (4–5 × 10^5^) were mixed with 400 μL of R1 buffer (GrisP) and 1 mM DTT and incubated at room temperature for 5 min. After adding 70% ethanol, the samples were transferred to an RNA mini spin column, washed, and treated with DNase I “in column” at room temperature for 15 min to remove contaminating DNA. RNA was eluted in RNase-free water (40 μL) and evaluated by Nanodrop DS 11 (DeNovix, DE, United States). The RNA of all the samples included in the study showed a 260/280 ratio of approximately 2.0 and a concentration ranging from 8 to 20 ng/μL.

### 2.4. qRT real-time PCR

DNase-treated RNA (100 ng) was reverse-transcribed into cDNA using the Improm-II Reverse Transcription System (Promega, WI, United States) according to the manufacturer’s protocol. In all reverse transcriptase reactions, a no-template control and another without the enzyme were included to monitor DNA contamination.

An amount of 2.5 ng of initial RNA in the RT reaction was used to quantitatively evaluate the transcriptional levels of the ENV gene of HERV-K, HERV-W and HERV-H and the gene expression of the pro-inflammatory cytokines IL-1β, IL-6, IL-10, TNF-α, MCP-1, IFN-α, IFN-β, IFN-γ, and IL-17 and its receptor IL-17RA, Toll-like receptor (TLR)-3, TLR-4 and TLR-7, and SARS-CoV-2 receptor ACE2 by real-time PCR (all primer pairs are listed in Supplementary Table S1).

The assays were performed in a Bio-Rad instrument (CFX96 Real-Time System, Bio-Rad, CA, United States) using SYBR Green chemistry (iTaq Universal SYBR green Supermix, Bio-Rad). To set up the real-time PCR assay, a serial dilution (10-fold) was performed to calculate efficiencies and correlation coefficients by the formula [efficiency = 10 (−1/slope)], and all primer pairs used showed an efficiency ranging from 0.96 to 0.98. Real-time PCR included forward and reverse primers (150 nM each) and 10 μL of 2X Fast QPCR Master Mix (SmoBio, Taiwan). The reaction was conducted for 1 cycle at 95°C for 3 min, then for 40 cycles at 95°C for 45 s, and at 60°C for 1 min. Each sample was analyzed in triplicate, and a negative control (without template) was included in each experiment to check out any possible contamination. The housekeeping beta-glucuronidase gene (GUSB) was used to normalize the results. Each experiment was completed with a melting curve analysis to confirm the specificity of amplification and the lack of any nonspecific product and primer dimer. Quantification was performed using the threshold cycle (Ct) comparative method: the relative expression was calculated as follows: 2^−[∆Ct (sample) − ∆Ct (calibrator)]^ = 2^−∆∆Ct^, where ∆Ct (sample) = [Ct (target gene) − Ct (housekeeping gene)] and ∆Ct (calibrator) was the mean ∆Ct of all SARS-CoV-2-negative samples. The comparison of GUSB Cts among the groups did not show significant differences (*p* = 0.98).

Beyond the diagnosis of SARS-CoV-2 infection evaluated at the Virology Unit of the PTV, SARS-CoV-2 N gene expression was carried out using the same methodological approach used for the other genes to better compare the data obtained. For this purpose, a specific primer for the N gene of SARS-CoV-2 (AAATTTTGGGGACCAGGAAC) at a concentration of 0.5 μM was used in a separate RT reaction to ensure the production of a specific cDNA. Real-time PCR for the N gene was conducted under the same conditions described above, and the results were expressed as 2^-∆Ct (sample) = [Ct (target gene) − Ct (housekeeping gene)]^.

### 2.5. Statistical analysis

Statistical analysis of groupwise expression levels was performed through the nonparametric Mann–Whitney test in the case of two independent samples or the Kruskal–Wallis test followed Bonferroni’s correction in the case of *n* independent samples. To identify associations between biomarkers in a multivariate manner, we performed a factor analysis followed by varimax rotation and Kaiser normalization. Factors were retained when associated with eigenvalues larger than one, and loadings were extracted through regression. The factor analysis was repeated separately in three groups: controls, SARS-CoV-2-positive only, and SARS-CoV-2-positive hospitalized (in the latter two groups, the N gene of SARS-CoV-2 was also included in the analyses). Data analyses were performed using the SPSS statistical software system (version 24.0 for Windows, United States), and statistically significant comparisons were considered when *p* < 0.05.

Moreover, to assess the strength of the correlations between the PCs identified in the group of hospitalized patients and the biochemical indicators, Spearman’s correlation analysis was conducted using an in-house developed MATLAB script.

### 2.6. Machine learning analysis

We employed a machine learning (ML) algorithm to explore the joint discrimination potential of all independent factors extracted from the previous analysis when distinguishing hospitalized vs. non hospitalized SARS-CoV-2-positive patients (43 patients, of whom 14 were hospitalized). This analysis was implemented in Python 3.6 using the scikit-learn python module (Pedregosa et al., 2011). Data were split randomly 100 times into training and test sets (70% vs. 30%) in a stratified manner. Classification was performed on each split by using extreme gradient boosting (Chen and Guestrin, 2016). For each training set, hyperparameter optimization was performed through a grid search in an inner 5-fold cross-validation fashion. After training the model, its performance was assessed for each test set by calculating the mean (across 100 repetitions) and standard deviation (SD) of the area under the curve (AUC) from the receiver operating characteristic curve, the accuracy, the sensitivity, the specificity, the f1-score (e.g., the harmonic mean of precision and recall) as well as the positive and the negative predicted values (PPV and NPV, respectively). To univocally rank the contribution of each factor to the final discrimination performance, we employed Shapley Additive explanations (SHAP) values on each training set (Lundberg and Su-In, 2017). We also analyzed each component’s contributions to classification performance at the single-patient level using SHAP partial dependence plots.

## 3. Results

### 3.1. Demographic characteristics, clinical status, hematologic, and biochemical profile of COVID-19 patients

The study included 63 nasopharyngeal and oropharyngeal swabs collected from March to May 2020 from individuals who attended the emergency room of Policlinico Tor Vergata, PTV (Rome, Italy) (Table 1A). Among these, 20 swabs were SARS-CoV-2-negative (mean age in years ± standard deviation, 70.6 ± 11.73; 14 males and 6 females) and 43 were positive (mean age in years ± standard deviation 68.56 ± 13.57; 29 males and 14 females). For statistical analyses, two groups of positive samples were analyzed: the first including 14 samples from individuals who were immediately hospitalized at the time of the swab result (hereafter referred to as hospitalized individuals, HOSPs; mean age in years ± standard deviation 66.33 ± 14.41; 8 males and 6 females) and the second including 29 samples of individuals who did not require hospitalization (hereafter referred to as not hospitalized individuals, not-HOSPs; mean age in years ± standard deviation 68.48 ± 11.77; 20 males and 9 females). The SARS-CoV-2-positive hospitalized patients were further grouped according to respiratory support received during hospitalization: 6 patients did not receive respiratory support (referred to as None Ox; mean age, in years ± standard deviation 68.33 ± 16.62; 3 males and 3 females), while 8 patients (mean age, in years ± standard deviation 64.13 ± 10.25; 5 males and 3 females) received respiratory support (referred to as Ox) such as nasal cannula/Venturi mask (NC/VMK), noninvasive ventilation (NIV/C-PAP) or invasive ventilation (orotracheal intubation, OTI). No statistically significant differences (*p* < 0.05) differences in age and sex were found between the patient groups.

**Table 1A:** Demographics of SARS-CoV-2-negative and -positive patients, categorized with respect to oxygen support at sampling.

	Negative	Positive	Positive			
			Not hospitalized	Hospitalized		
				None Ox	Ox	Tot
Number	20	43	29	6 (42.85%)	8 (57.14%)	14
Age (Mean ± SD)	70.6 ± 11.73	68.5 ± 13.57	68.48 ± 11.77	68.33 ± 16.62	64.13 ± 10.25	66.33 ± 14.41
Male	14	29	20	3	5	8
Female	6	14	9	3	3	6

None Ox, No oxygen support; Ox, oxygen support at hospitalization.

HOSPs showed radiological signs of monolateral interstitial pneumonia (MiP) in 6 cases and bilateral (BiP) in 8 cases (Table 1B). The hematological and biochemical parameters of HOSPs, evaluated at the time of the swab collection were also reported in Table 1B. Compared with the reference values, a reduction in the fraction of inspired O_2_ (FiO_2_) and changes in some biochemical parameters, such as coagulation (fibrinogen, D-dimer, lactate dehydrogenase (LDH)), liver markers (blood urea nitrogen, BUN, and aspartate aminotransferase, AST), the inflammatory marker reactive C protein (CRP), the cardiac alteration of B-type natriuretic peptide (BNP) and slight lymphopenia were observed.

**Table 1B:** Clinical status, hematological profile, lymphocyte subpopulations and biochemical parameters of hospitalized COVID-19 patients.

*Clinical status*			
Pneumonia**		None	0
		MiP	6
		BiP	8
*Hematological profile*			
		Reference values	Values at sampling
Red blood cells (10^6^/μL)		4.40–6.00	4.1 ± 1.1
Hemoglobin (g/dL)		13.00–18.00	11.6 ± 2.9
White blood cells (10^5^/μL)		4.30–10.80	18 ± 33.6*
Neutrophils	Abs count (10^3^/uL)	2.1–6.5	15.8 ± 34.4
	%	40–75	**76.8 ± 15.5**
Lymphocytes	Abs count (10^3^/uL)	10–45	1.3 ± 0.7
	%	20–45	15.9 ± 13
Monocytes	Abs count (10^3^/uL)	0.27–0.92	0.9 ± 1.2
	%	3.4–11	6.4 ± 2.1
Eosinophils	Abs count (10^3^/uL)	0.04–0.45	0.04 ± 0.1
	%	0–7	0.5 ± 0.1
Basophils	Abs count (10^3^/uL)	0.00–0.20	0.03 ± 0.03
	%	0–1.5	0.4 ± 0.3
*Lymphocyte subpopulations*			
CD3+	Abs count (cell/mm^3^)	690–2,540	110.6 ± 819.2
	%	55–84	76.6 ± 13.2
CD3 + CD4+	Abs count (cell/mm^3^)	31–60	**601.3 ± 398.8**
	%	410–1,590	48 ± 7.1
CD3 + CD8+	Abs count (cell/mm^3^)	190–1,140	491.6 ± 451.4
	%	13–41	30.4 ± 13
CD19+	Abs count (cell/mm^3^)	90–660	168.9 ± 100.7
	%	5–25	14.4 ± 10.8
*Biochemical parameters*			
Fibrinogen (mg/dL)		200–400	**511.1 ± 262.2**
Antithrombin III (%)		75–128	101.5 ± 30.8
D-Dimers (ng/mL)		0–500	**1314.9 ± 1318.1**
Glucose (mg/dL)		70–100	**137 ± 112.7**
BUN (mg/dL)		15–40	**49.3 ± 28.8**
LDH (U/L)		125–220	**288.5 ± 123.1**
ALT (U/L)		0–55	44.4 ± 60.2
AST (U/L)		5–34	**42.2 ± 43.6**
Reactive C Protein (CRP) (mg/L)		0–5	**58.2 ± 74.9**
Lipase (U/L)		8–78	**35 ± 19.2**
Amylase (UI/L)		20–160	77.3 ± 43.1
FiO2 (%)		30–40	**10.56 ± 12.65**
PT (%)		70–130	75.6 ± 13.3
PT (sec)		10–13	**14.2 ± 2**
BNP (pg/mL)		<100	**177 ± 176.3**
hs-Troponin (ng/L)		≤15.6	**15.9 ± 23.6**

**None, no pneumonia; MiP, Monolateral or minimal interstitial pneumonia; BiP, bilateral interstitial pneumonia. Number in bold: value outside the reference values.

### 3.2. The expression of HERVs and inflammatory mediators was found to be modulated in SARS-CoV-2-positive swabs

**Figure 1:**
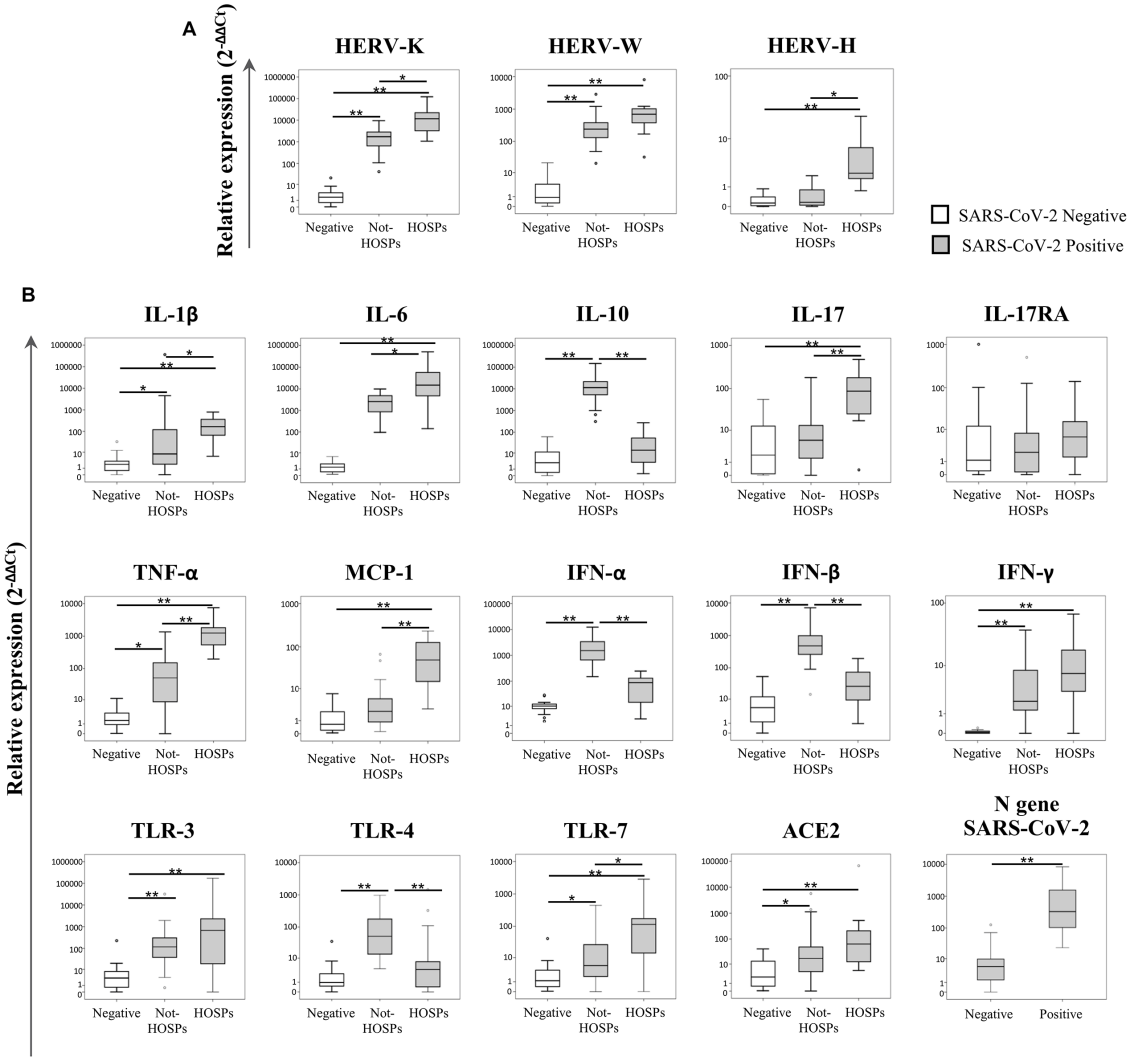
Expression levels of the ENV gene of HERV-K, HERV-W and HERV-H, inflammatory mediators and SARS-CoV-2 infection-related genes in nasopharyngeal and oropharyngeal swab samples. The transcriptional levels of the ENV gene of HERV-K, HERV-W and HERV-H **(A)**, cytokines (IL-1β, IL-6, IL-10, IL-17, TNF-α, MCP-1, IFN-α, IFN-β, and IFN-γ), inflammatory mediators (IL-17RA and TLRs), ACE2 and the N gene of SARS-CoV-2 (ACE2 and N gene) **(B)** were analyzed by qRT-PCR in 20 SARS-CoV-2-negative and 43 SARS-CoV-2-positive swab samples. Among SARS-CoV-2-positive swabs, 29 samples came from individuals who did not require hospitalization (not-HOSPs) and 14 samples came from individuals who were immediately hospitalized at the time of the swab result (HOSPs). The results are represented as box plots (white box plots for negative swab samples, gray box plots for positive swab samples), depicting mild (black dot), and extreme outliers (asterisk) for each group. Group-wise differences were examined using the nonparametric Kruskal–Wallis test (statistical significance was defined when *p* < 0.050).

The transcriptional levels of the ENV gene of HERV-K, HERV-W and HERV-H, inflammatory mediators, and SARS-CoV-2 infection-related genes were analyzed in 20 SARS-CoV-2-negative and 43 SARS-CoV-2-positive swab samples (29 from not-HOSPs and 14 from HOSPs patients). The data obtained are represented in Figure 1 as box plots; median values, interquartile range, and results of the Kruskall-Wallis test are reported in Supplementary Table S2.

The expression of the ENV gene of both HERV-K and HERV-W (Figure 1A) was higher in positive swabs (regardless of the patient’s hospitalization) than in negative swabs (*p* < 0.001). Among the SARS-CoV-2-positive samples, the ENV gene of HERV-K was more highly expressed in hospitalized individuals than in individuals who were not hospitalized (*p* = 0.021), while HERV-H was highly expressed only in positive swabs from hospitalized individuals with respect to negative swabs and positive swabs from not-HOSPs (*p* < 0.001).

In SARS-CoV-2-positive swabs higher expression levels of IL-1β (*p* < 0.001), IL-6 (*p* < 0.001), TNF-α (*p* = 0.003), IFN-γ (*p* < 0.001), TLR-3 (*p* < 0.001) and TLR-7 (*p* = 0.030) were found when compared to negative swabs (Figure 1B). Among SARS-CoV-2-positive swab samples, IL-10, IFN-α, INF-β and TLR-4 expression was significantly higher in individuals who had not been hospitalized than in hospitalized and in negative swabs (respectively IL-10 *p* < 0.001; IFN-α *p* < 0.001; IFN-β *p* < 0.001; TLR-4 *p* = 0.004). Conversely, higher expression levels of IL-1β, IL-6, IL-17, TNF-α, MCP-1 and TLR-7 were observed in swabs from individuals who were hospitalized than in those who were not hospitalized (respectively IL-1β *p* = 0.033; IL-6 *p* = 0.040; IL-17 *p* = 0.001; TNF-α *p* = 0.001; MCP-1 *p* = 0.001; TLR-7 *p* = 0.040). ACE2 was more highly expressed in SARS-CoV-2-positive than in negative swab samples (*p* = 0.035). The N gene of SARS-CoV-2 was more highly expressed in hospitalized individuals than in those who were not hospitalized (*p* = 0.001). No statistically significant differences were revealed for IL-17RA for any of the comparisons.

### 3.3. Factor analysis demonstrated HERVs and inflammatory mediators as the main carriers of information only in SARS-CoV-2-positive hospitalized patients

To investigate the complex interplay of HERVs and inflammatory mediators, a principal component (PC) analysis was performed separately in the three groups: SARS-CoV-2-positive (HOSP and not-HOSP) and in negative (Table 2). The factor analyses performed on the SARS-CoV-2-positive hospitalized patients yielded 5 factors explaining 90% of the total variance (Table 2): component 1, which explained 38% of the total variance, mainly loaded on the N gene of SARS, IL-10, HERV-W, IL-6 and TNF-α while component 2 (~27% of the total variance) heavily loaded on TLR-3, TLR-7, IL-17RA, and HERV-K. Components 3, 4, and 5 which cumulatively explained ~25% of the variance, loaded on: TNF-α, IL-1β, and IFN-β (component 3); IL-17, IFN-γ, and TLR-4 (component 4); and on ACE2 and HERV-H (component 5). In the case not-HOSPs SARS-CoV-2-positive swab samples, ~75% of the total variance was explained by 7 factors (Table 2): component 1, which carried a large share of the information (~26%), is heavily loaded on IFN-β, TRL-4, and TNF-α; component 2 loaded on IL-17RA, HERV-H and IFN-γ (~12%); component 3 loaded on HERV-W and TLR-7 (~10%); component 4 loaded on HERV-K and ACE2 (~9%), component 5 loaded on MCP-1 and IL-6 (~7%); component 6 loaded on the N gene of SARS-CoV-2 (~6%) and component 7 (~6%) loaded on IL-6, IL-1β, TLR-3. As shown in Table 2, the same analysis performed on the SARS-CoV-2-negative samples showed that 5 factors explained 88% of the total variance: the first component (~51% of the total variance) loaded on IL-17RA, TNF-α TLR-3, TLR-4, TLR-7, IL-10, MCP-1, and HERV-W, although these genes were expressed at very low level. The other four components explained ~13%, ~12%, ~7%, and ~ 6% of the variance and were mainly loaded on IL-17, TNF-α; ACE2, HERV-H; IFN-β; and IL-1β, respectively.

**Table 2:** Principal components (PC) and hierarchical clustering of HERVs, inflammatory mediators, and SARS-CoV-2 infection-related genes in SARS-CoV-2-positive (HOSP and NOT-HOSP) and -negative nasopharyngeal and oropharyngeal swabs.

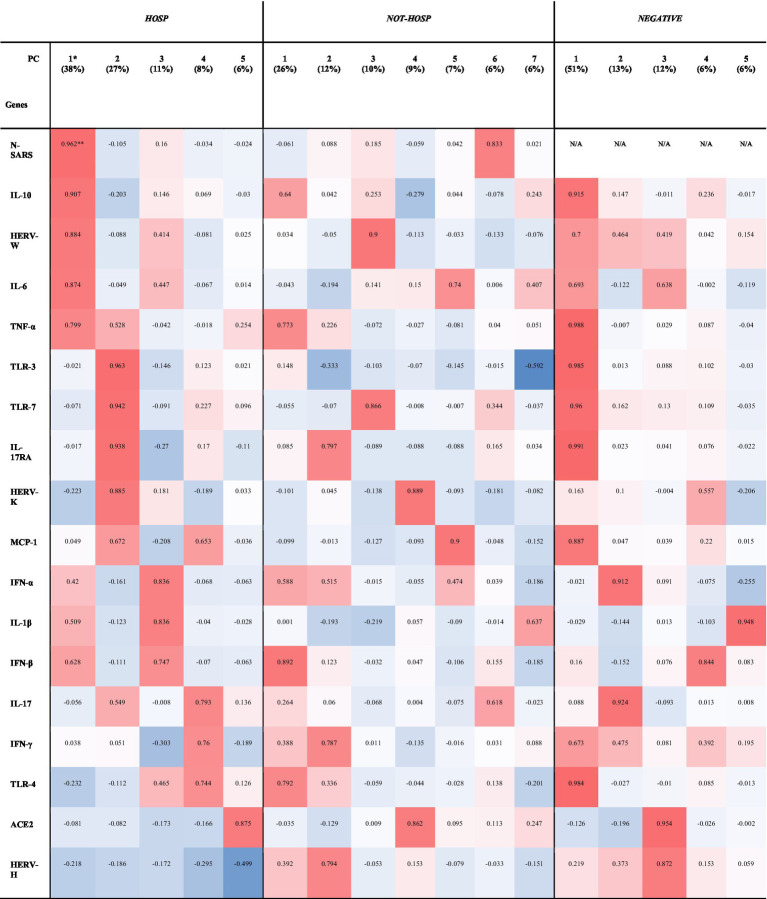

*In parentheses the percentage of the total variance explained by the component. **Parameters in blue are inversely associated while those in red are directly associated.

### 3.4. Machine learning results suggest HERVs as predictive elements for hospitalization

To evaluate potential predictive biomarkers for hospitalization of SARS-CoV-2-positive patients a machine learning model based on extreme gradient boosting and explain ability techniques was used.

**Figure 2:**
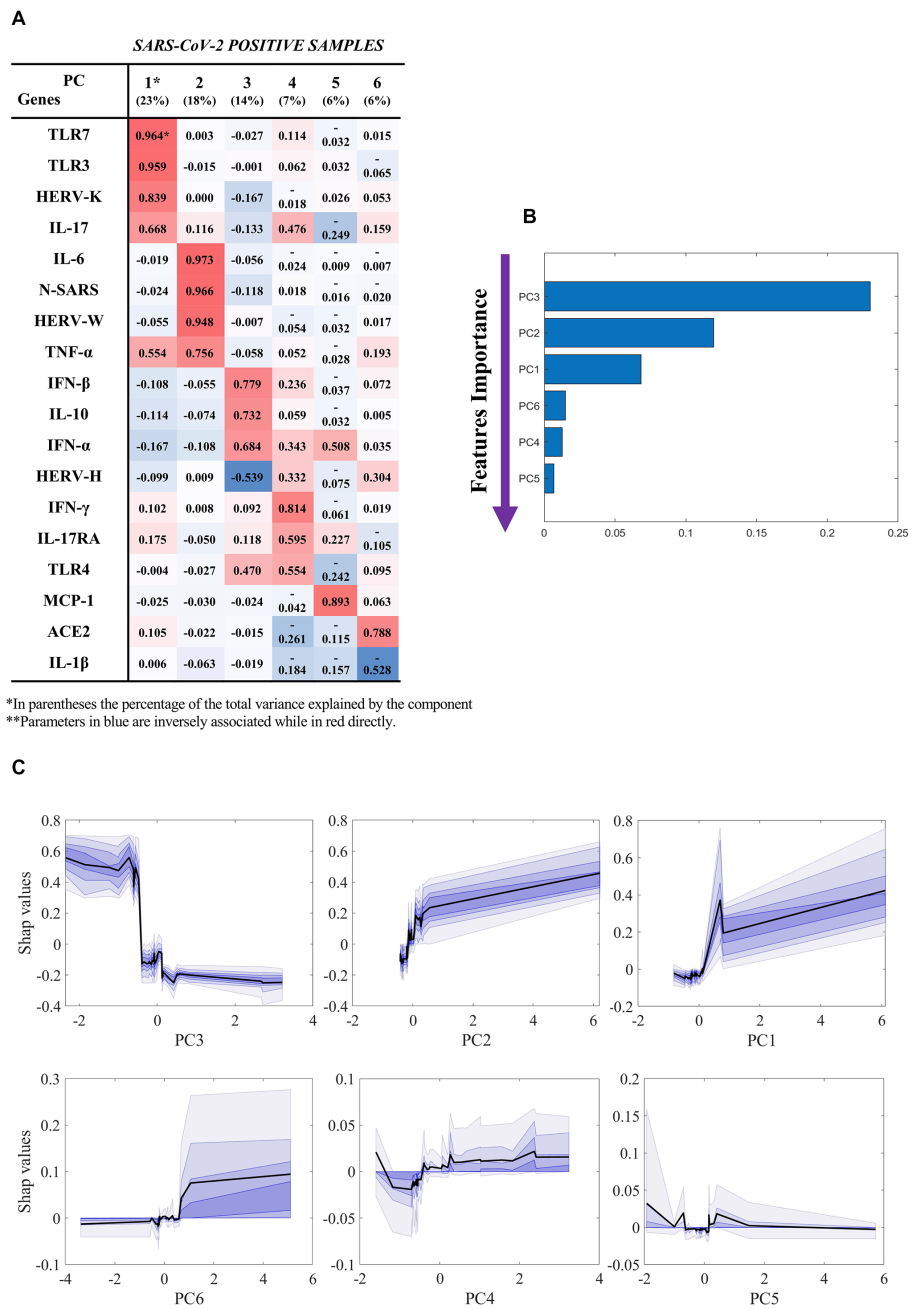
Results of the machine learning model (hospitalized *vs* not hospitalized SARS-CoV-2-positive). Based on the results of factor analysis. **(A)** Cumulative variance and single loadings computed through factor analysis; **(B)** feature importance ranking derived through SHAP values; **(C)** SHAP dependence plots for each component, showing median and confidence intervals across 100 repetitions, in black and violet, respectively. Summary of classification performance: AUC 0.85 ± 0.12; Accuracy 0.90 ± 0.08; Sensitivity 0.98 ± 0.05; Specificity 0.73 ± 0.22; f1-score 0.80 ± 0.18; PPV 0.94 ± 0.13; NPV 0.90 ± 0.08.

Figure 2 reports the results of our classification model (SARS-CoV-2-positive hospitalized *vs* not hospitalized), which was based on the components extracted by factor analysis of SARS-CoV-2-positive samples (Figure 2A). By using the six components, the machine learning model was able to classify hospitalized *vs* not hospitalized SARS-CoV-2-positive patients with an AUC of 0.85 ± 0.12, accuracy of 0.90 ± 0.08, sensitivity of 0.98 ± 0.05, specificity of 0.73 ± 0.22, f1-score of 0.80 ± 0.18, PPV of 0.94 ± 0.13 and NPV of 0.90 ± 0.08. The unique contribution of each component to this performance is reported in Figure 2B, where larger values indicate a larger contribution to the final prediction. The most important feature is component 3, which mostly loaded on IFN-β, IL-10, IFN-α, and TLR-4. As shown by the partial dependence plots (Figure 2C), which depict the importance of each components as a function of the value of the component itself, the importance of this component in the classification remained stable as the component value increased within a narrow interval, after which its importance decreased abruptly and then remained stably low. In contrast, the importance of components 1 (mainly loaded on HERV-K, TLR-3, TLR-7, and IL-17), 2 (mainly loaded on HERV-W, IL-6, TNF-α, and N gene of SARS-CoV-2) and 6 (mainly loaded on ACE2) increased as the value of the component increased, while the effect of components 4 and 5 were stable across all individuals.

### 3.5. In hospitalized SARS-CoV-2-positive patients HERV-K and HERV-W expression correlates with markers of coagulation and T-cell-mediated immune response

Spearman’s correlation analysis using data extracted from factor analysis (see Table 2, column “*HOSP*”) showed several correlations between the components identified by factor analysis and biochemical markers in hospitalized patients (Table 3). Considering the first two components, containing HERV-W and HERV-K, a positive correlation was found with the plasmatic level of fibrinogen (Rho 0.822, *p* = 0.014 and Rho 0.623, *p* = 0.017, respectively). Moreover, the second component negatively correlated with eosinophil cell percentage (Rho −0.577, *p* = 0.031), prothrombin time percentage and (Rho −0.543, *p* = 0.045), absolute count of CD3 (Rho −0.846, *p* = 0.026) and CD4 (Rho −0.821, *p* = 0.034), while positively correlated with CD19 percentage (Rho 0.786, *p* = 0.048).

**Table 3:** Results of Spearman’s correlation analysis.

	PC1	PC2	PC3	PC4	PC5
	N-SARS, IL-10, HERV-W, IL-6, TNFa	TLR-3, TLR-7,IL-17RA, HERV-K	TNF-α, IL-1β, IFN-β	IL-17, IFN-γ, TLR-4	ACE2, HERV-H
*P*/*F* ratio				Rho −0.706 *p* = 0.039	
FiO_2_ (%)			Rho −0.857 *p* = 0.008		
EO (%)		Rho −0.577 *p* = 0.031			
PT (%)		Rho −0.543 *p* = 0.045	Rho −0.645 *p* = 0.013		
PT (Sec)			Rho 0.607 *p* = 0.021		
Fibrinogen (mg/dL)	Rho 0.822 *p* = 0.014	Rho 0.623 *p* = 0.017			
Glucose (mg/dL)					Rho −0.608 *p* = 0.024
BUN (mg/dL)					Rho −0.771 *p* = 0.002
CPR (mg/L)			Rho 0.595 *p* = 0.025		
BNP (pg/mL)			Rho −0.821 *p* = 0.034		Rho −0.857 *p* = 0.043
hs-Troponin (mg/L)			Rho −0.781 *p* = 0.005		Rho −0.616 *p* = 0.043
CD3+ (%)		Rho −0.847 *p* = 0.025			
CD3 + CD4+ (count)		Rho −0.821 *p* = 0.034			
CD3+ CD8+ (count)				Rho −0.821 *p* = 0.034	
CD19 (%)		Rho 0.786 *p* = 0.048			

### 3.6. The expression of HERV-W ENV in SARS-CoV-2-positive swab samples stratifies oxygen need in COVID-19 patients

**Figure 3:**
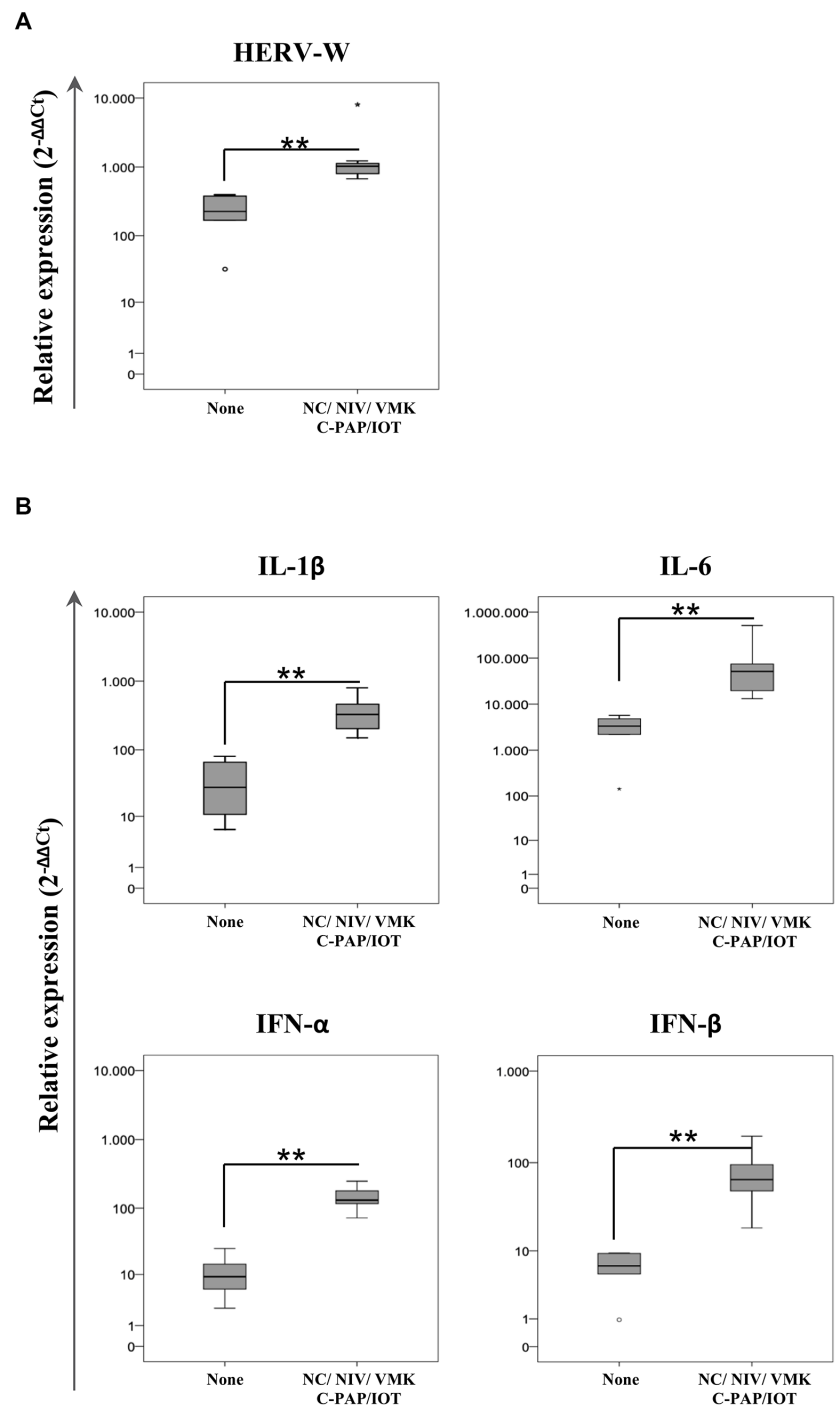
Relative expression of HERV-W ENV **(A)** and pro-inflammatory mediators **(B)** in positive swab samples from hospitalized COVID-19 patients stratified by oxygen support. HOSPs SARS-CoV-2-positive swabs were stratified according to the respiratory needs of patients during hospitalization: no oxygen support needed (None; *n* = 6) and oxygen support with a nasal cannula or Venturi mask (NC/VMK) and by non-invasive ventilation, continuous positive airway pressure or orotracheal intubation (NIV/C-PAP/OTI; *n* = 8). The mRNA expression of HERV-W ENV and pro-inflammatory mediators (IL-1β, IL-6, IFN-α, and IFN-β) are represented as box plots. The non-parametric Kruskall-Wallis test was used to compare groups and statistically significant values were considered when *p* < 0.050.

Using the information on the required oxygen supply during the course of the disease, positive swab samples from individuals who were hospitalized were included into two groups according to their respiratory outcome: no oxygen support (none) and oxygen support (regardless of the type of oxygen support with NC/VMKs and oxygen support by NIV/C-PAP/OTI). Statistical analysis demonstrated that HERV-W ENV expression was higher in the group of individuals who needed oxygen support in comparison to the “none” group (*p* = 0.003) (Figure 3; Supplementary Table S3). The expression of IL-1β, IL-6, IFN-α and IFN-β was also higher in the oxygen support group (*p* = 0.003).

### 3.7. *In vitro* stimulation with SARS-CoV-2 spike protein increases HERV expression in a hypopharyngeal carcinoma cell line ahead of inflammatory markers

**Figure 4:**
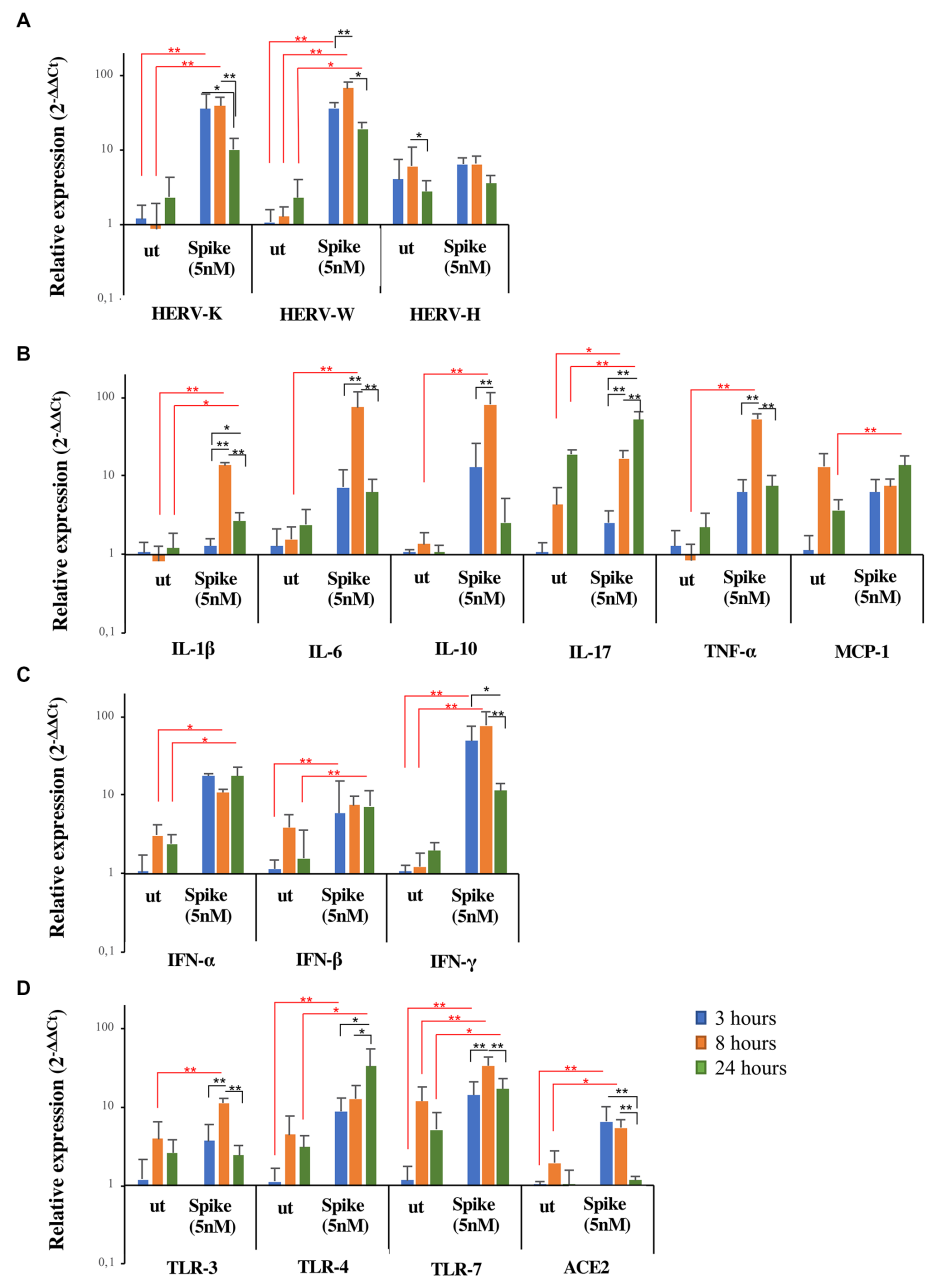
Expression of HERVs and inflammatory markers in FaDu cells after *in vitro* stimulation with SARS-CoV-2 spike protein. FaDu cells were stimulated with SARS-CoV-2 spike protein (5 nM) for 3, 8 and 24 h. HERVs **(A)**, cytokines **(B)**, interferons **(C)** and receptors **(D)** mRNA levels, obtained by qRT-PCR analysis, were represented as mean ± standard deviation. For comparisons non parametric Kruskal–Wallis test was utilized. Red lines and asterisks outline differences between untreated (ut) and treated with spike protein, while black lines and asterisks delineate differences between spike treatments at different times of exposure.

To clarify the kinetics of activation of HERV-H, HERV-K, and HERV-W and inflammatory mediators by SARS-CoV-2, a hypopharyngeal carcinoma cell line (FaDu) was stimulated *in vitro* for 3, 8, and 24 h with the spike protein. The results are represented as the mean value ± standard deviation in Figure 4. The *in vitro* exposure to spike protein significantly induced the expression of HERVs and cytokines compared to untreated FaDu cells, although with different kinetics depending on the gene analyzed (see Supplementary Tables S4, S5 for mean values and results of statistical analysis of nonparametric Kruskal–Wallis test). Indeed, after spike treatment, HERV-K, HERV-W, IFNs, and ACE2 reached the peak of expression already at 3 h, while IL-1β, IL-6, IL-10, IL-17, TNF-α, and TLRs showed the highest levels between 8 h and 24 h. Concerning HERV-H and MCP-1, no differences in the expression levels between untreated and treated cells were found.

## 4. Discussion

The role of HERVs in innate immunity and human diseases has been widely reported, as amplified HERV transcriptional activity has been described in several pathological conditions, including autoimmunity, cancer, and infectious diseases (Wang et al., 2015; Kassiotis and Stoye, 2017; Charvet et al., 2018; Dai et al., 2018). Herein, we evaluated the impact of SARS-CoV-2 infection on the transcriptional activation of HERVs and induction of inflammatory mediators at the level of the naso-oropharyngeal mucosal tissue in individuals attending the emergency unit at the early stage of the infection, to determine HERV involvement in COVID-19 pathogenesis onset and to assess a complex expression profile for an early prediction of severity and disease outcome. The results demonstrated that activation of HERVs occurs at the site of SARS-CoV-2 entry into the nasal mucosa, where a significant increase in HERV-K, HERV-W and HERV-H transcriptional activity was found in positive individuals with respect to the negative individuals. Interestingly, stratifying the SARS-CoV-2 study cohort according to the need for hospitalization, higher levels of HERV-K and HERV-H expression were found in individuals who underwent hospitalization. HERVs can be activated by exogenous viruses such as the herpesviruses, hepatitis B virus, the human immunodeficiency virus-1 and influenza A virus (Toufaily et al., 2006; Li et al., 2014; Charvet et al., 2018; Leung et al., 2018), and their products (DNA, RNA, and proteins) may be recognized by different pattern recognition receptor (PRRs), inducing an innate immune response and the establishment of the antiviral state, similar to that of exogenous viruses (Rolland et al., 2006; Uleri et al., 2014; Chiappinelli et al., 2015; Madeira et al., 2016; Wang et al., 2018). It is also known that HERVs are responsive also to inflammatory transcription factors contributing to the pathobiology of HERV-associated inflammatory diseases (Manghera and Douville, 2013; Manghera et al., 2016).

In the context of COVID-19, our research group and others have described the activation of HERVs in various tissues, including bronchoalveolar lavages, tracheal aspirates and blood samples, from SARS-CoV-2-positive patients. Specifically, by transcriptome analysis, transposable elements including HERVs, were found to be dysregulated in the bronchoalveolar lavage fluid of COVID-19 patients and in senescent human bronchial epithelial cells *in vitro*, suggesting an explanation for the differences in disease severity with the age of patients (Kitsou et al., 2021; Marston et al., 2021). Even in tracheal aspirates, higher levels of HERV-K were found in critically ill and deceased patients in comparison to nasopharyngeal swabs from mild cases and tracheal aspirates from non-COVID patients (Temerozo et al., 2022). Interestingly, parallel to an increase in HERV transcripts in blood samples from COVID-19 patients (Balestrieri et al., 2021; Guo et al., 2022), we demonstrated that HERV-W ENV protein expression was high in the leukocytes and correlated with the markers of T-cell differentiation and exhaustion and cytokine levels, suggesting the involvement of ENV protein in COVID-19 immunopathology (Balestrieri et al., 2021).

Besides HERV induction, we also showed concomitant higher levels of several pro-inflammatory cytokines, chemokines and TLRs (IL-1β, IL-6, IL-17, TNF-α, MCP-1, TLR-3, and TLR-7) and lower levels of type I IFNs, IL-10, and TLR-4 in individuals who underwent hospitalization. These inflammatory mediators were already described to be major actors in the cytokine storm in COVID-19 patients, playing an important role in the airway immune response. Elevated IL-1β and IL-6 responses have been associated with disease severity (Huang et al., 2020; Liao et al., 2020; Qin et al., 2020; Ravindra et al., 2020; Zhang X. et al., 2020; Zhou et al., 2020), suggesting that IL-1β and/or IL-6 may be key drivers of pathology in severe COVID-19. These markers have high potential, rapidly increasing inflammatory cytokine/chemokine responses in the upper airway, likely predicting subsequent pathologic events in the lower airway associated with acute respiratory distress syndrome (Glaser et al., 2019). TLR signaling plays a key role in the innate immune system, orchestrating inflammatory responses, by the production of type I interferon co-stimulatory molecules or the induction of a pro-inflammatory cytokine cascade (Chen et al., 2007). The antiviral response by type I IFN is activated early in COVID-19 patients and can potentially predict the disease outcome. Considering the major role of two interferons (IFN-α/β), which have a broad-spectrum of antiviral activities against RNA viruses and act by inducing an antiviral state favoring the adaptive immune response (Mantlo et al., 2020; Minutolo et al., 2021), and the TLR-4, which is involved in molecular pattern recognition and interacts with the spike protein (Zhao et al., 2021a), our data suggest their involvement in antiviral protection mechanisms in patients who did not need to be hospitalized. In contrast, a deficient interferon response associated with low levels of TLR-4 has been proposed as one of the relevant mechanisms prompting severe manifestations of COVID-19 (Zhang Q. et al., 2020; Contoli et al., 2021; Masood et al., 2021). It is also important to highlight that since SARS-CoV-2 infection at some level inhibits the interferon response and a significant inflammatory phase occurs (Yuen et al., 2020), the identification and modulation of factors involved in cytokine storm should be further studied (Matteucci et al., 2020). From this perspective, the presence of type I IFN autoantibodies has been demonstrated to be a strong predictor of death in COVID-19 patients (Bastard et al., 2020; Manry et al., 2022). Notably, in a recent study, the presence of higher levels of autoantibodies against IFN-α and IFN-ω were found in intensive care unit (ICU) patients with life-threatening COVID-19 than in subjects with mild COVID-19 and healthy subjects, suggesting that the autoantibodies against IFNs may result in a direct damage to the host rather than a protection against infection (Bastard et al., 2021; Casanova and Abel, 2022; Simula et al., 2022). Moreover, high levels of antibodies against the HERV-W ENV epitope were found in the ICU patients, and exhibited a strong correlation with type I IFN autoantibodies, particularly anti-IFN-α, suggesting that the HERV activation and the deregulation of IFNs have participating roles in the immunopathology of COVID-19 (Simula et al., 2022). In addition, the deficient IFN-α levels have been associated with increased IL-10 expression in blood samples from patients progressing to severe outcomes in COVID-19 conditions (Contoli et al., 2021), while in the present study, we found low levels of IL-10 in patients who underwent hospitalization, suggesting that its expression may indicate the need for hospitalization of COVID-19 patients. This discrepancy could be due to the different tissues analyzed, which reflect two distinct phases of the immune response to the infections, on the one side, the first line of defense and on the other side, the systemic one. Notably, in COVID-19 patients, excessive immune activation and subsequent cytokine storm occur, and to prevent damage to host tissues, immunoregulatory cytokines, such as IL-10, are produced to downregulate the expression of pro-inflammatory cytokines (Zhang and Bastard, 2022).

The interplay between HERVs and innate and inflammatory pathways in COVID-19 severity was corroborated by the factor analysis. In fact, the analysis allowed the identification of a specific component that connects HERW-W with N-SARS, IL-10, IL-6, and TNF-α and another component outlined HERV-K with TLR-3, TLR-7, MCP-1, and the IL-17RA in individuals who underwent hospitalization. Conversely, in SARS-CoV-2-positive swab samples of those individuals who did not require hospitalization, the most representative component was mostly loaded on cytokines such as IL-10, TNF-α, IFN-α, IFN-β, and TLR-4.

Intriguingly, comparing the expression profiles of SARS-CoV-2-positive swab samples of individuals who underwent hospitalization to samples from individuals who did not by means of machine learning analysis, we were able to identify highly significant components defining a complex expression profile predictive of the need for hospitalization at the early stage of the infection. The components with the higher predictive value were loaded on HERV-W, IL-6, N-SARS and TNF-α, or on HERV-K TLR-7, TLR-3, and IL-17. A third important predictive component loaded on IFN-β, IL-10, IFN-α, HERV-H, and TLR-4 was indeed differentially expressed in patients who needed hospitalization.

Notably, in hospitalized patients the complex expression profile including HERV-W, N-SARS, IL-6, IL-10, and TNF-α positively correlates with fibrinogen, while the component loaded on TLR-3, TLR-7, IL-17RA, and HERV-K correlates negatively with prothrombin (%), D-dimer, and CD4 T cells (%) and positively with fibrinogen and CD19 B cells (%), suggesting the involvement of HERVs in the pathogenesis of COVID-19. In line with our findings, several reports described the overproduction of IL-6, TNF-α, and IL-1β (Tay et al., 2020), leukopenia and coagulopathy, marked by platelet activation and high D-dimer levels, accounting for the development of the more severe forms of the COVID-19 (Terpos et al., 2020). We have already demonstrated that the expression of HERV-W protein was associated with several clinical and immunological alterations in COVID-19 patients, correlating with markers of inflammation, including cytokine expression, T-cell differentiation and functional exhaustion (Balestrieri et al., 2021).

Severe forms of COVID-19 are also characterized by oxygen requirements, ranging from oxygen supplementation *via* a face mask to intubation and mechanical ventilation, and are supported by several risk factors including older age, hypertension, diabetes, and obesity (Argenziano et al., 2020). In critically ill patients, higher HERV-K levels were associated with early mortality in the intensive care unit (Temerozo et al., 2022), and we have already shown that high HERV-W protein expression in CD4 T lymphocytes was found to be predictive of the need for oxygen support during hospitalization (Balestrieri et al., 2021). In the present study, we show that HERV-W is already high in the nasal mucosa and again reflects the need for oxygen during hospitalization, representing a potential genomic biomarker of the respiratory outcome of patients.

Taken together, the current findings support HERV activation as a contributing factor in COVID-19 immunopathology, picturing a close interplay with the immune response. HERV transcription could lead to the release of pathogen-associated molecular patterns, which, by interacting with sensors of innate immunity, could evoke the production of inflammatory mediators, contributing to the cytokine storm. On the other side, the inflammatory mediators induced by HERVs or other triggers could, in turn, further increase HERVs activity (Hurst and Magiorkinis, 2015; Gröger and Cynis, 2018; Balestrieri et al., 2019). Moreover, other groups demonstrated *in vitro* that in human and animal cells the S protein of SARS-CoV-2 interacts with host receptors TLR2, TLR4, and ACE2 to activate inflammatory immune responses (Lan et al., 2020; Khan et al., 2021; Shirato and Kizaki, 2021; Zhao et al., 2021b).

We have previously conducted an *in vitro* study using PBMCs from healthy donor demonstrating the induction of the pathogenic HERV-W ENV protein expression by the exposure to SARS-CoV-2 spike protein, which occurs before the expression of IL-6 (Balestrieri et al., 2021). Here we showed that the stimulation of an epithelial cells line from hypopharyngeal carcinoma leads to an early activation of HERV expression followed by an increase in several pro-inflammatory cytokines, suggesting early HERV activation consistent with its potential role in the inflammatory process related to infectious diseases.

Our study pictures a specific moment in the pandemic. The samples collected derived from patients arriving in emergency rooms of our hospital during the first COVID-19 wave, when in Italy the access for swabbing mainly concerned elderly subjects with risk for SARS-CoV-2 infections, while almost precluded for all the other diseases. Both HERVs and the inflammatory response may be activated also in other diseases, such as cancer, autoimmune, neurological, and neuropsychiatric diseases, some of which are recognized as comorbidities in COVID-19. Moreover, HERV activity, as well as the immune response, depends and varies according to the age. Therefore, the presence of comorbidities in our groups could contribute to the activation of HERVs and the inflammatory response in both SARS-CoV-2 positive or negative individuals included in our study. Nevertheless, all the analyzed samples are homogenous in terms of age and sex since, hence may have similar comorbidities. Among these patients, the fact that HERVs have been found expressed at high levels in positive samples, particularly in those who have been then hospitalized, suggests the infection as a triggering factor for the activation of HERVs and the inflammatory response. Moreover, we are aware that the development of genomic biomarkers is a multiphase and iterative process that begins with the identification of biomarkers in biological samples, and, not as done in this study, requires a subsequent phase of analytical validation and qualification for its possible use in clinical practice.^1^ Further study may be warranted to clarify if and how different variants of the virus impact HERV reactivation and how this interaction affects the COVID-19 severity and patient outcome.

The SARS-CoV-2 pandemic highlighted the need to define the determinants at the basis of disease pathogenesis and to identify genomic biomarkers predictive of the infection evolution, with the aim of defining personalized drug treatment. The current study demonstrated the activation of HERVs and mediators of the innate immune response in the initial site and at the early stage of SARS-CoV-2 infection. Thus, our findings sustain the interplay among HERVs and inflammatory mediators in the early response to SARS-CoV-2 infection and picture a complex profile potentially useful as a predictive biomarker of COVID-19 severity and patient outcome. In addition, the activation of HERVs as a contributing factor in COVID-19 immunopathology opens novel therapeutic opportunities targeting HERVs in this specific clinical setting.

## Data availability statement

The original contributions presented in the study are included in the article/supplementary material, further inquiries can be directed to the corresponding author.

## Ethics statement

The studies involving human participants were reviewed and approved by COVID_SEET prot.7562/2020, Tor Vergata University/Hospital. The patients/participants provided their written informed consent to participate in this study.

## Author contributions

ClM, EB, and SG conceived the study. VP, MF, MG, and ChM conducted the experimental work. MI and CR performed the acquisition of clinical data. MA, LS, and PR employed for clinical patients and sample management. NT, AC, CC, MM, FA, AM, and EB performed the data analysis. GN, EG, GR, and PSV revised manuscript critically for important intellectual content. CC, PSV, EB, and ClM wrote original draft. All authors contributed to the article and approved the submitted version.

## Funding

VP, MF, and CC were supported by the HERVCOV project funded by the HORIZONHLTH-2021-DISEASE project (Personalized medicine and infectious disease: understanding the individual host response to virus) of the European Commission under the Horizon Europe Framework Program. G.A.101057302.

## Conflict of interest

The authors declare that the research was conducted in the absence of any commercial or financial relationships that could be construed as a potential conflict of interest.

The reviewer TPH declared a past collaboration with the author ClM to the handling editor.

## Publisher’s note

All claims expressed in this article are solely those of the authors and do not necessarily represent those of their affiliated organizations, or those of the publisher, the editors and the reviewers. Any product that may be evaluated in this article, or claim that may be made by its manufacturer, is not guaranteed or endorsed by the publisher.

## Footnotes

^1^https://www.ema.europa.eu/en/news/european-medicines-agency-seeks-views-genomic-markers-medicine-development (Accessed January 30, 2023).
