# Supplementary material for: Expression profile of HERVs and inflammatory mediators detected in nasal mucosa as a predictive biomarker of COVID-19 severity
Source: Front Microbiol. 2023 May 22;14:1155624. doi: 10.3389/fmicb.2023.1155624 (PMC10239953; doi:10.3389/fmicb.2023.1155624)
Supplement: Supplementary file 1 [file Table_1.docx]

Supplementary Material

Expression Profile of HERVs And Inflammatory Mediators Detected In Nasal Mucosa As a Predictive Biomarker of COVID-19 Severity

Vita Petrone ^1†^, Marialaura Fanelli ^1†^, Martina Giudice ^1^, Nicola Toschi ^2,3^, Allegra Conti ^2^, Christian Maracchioni ^1^, Marco Iannetta ^4^, Claudia Resta ^5^, Chiara Cipriani ^1^, Martino Tony Miele ^1^, Francesca Amati ^2^, Massimo Andreoni ^4^, Loredana Sarmati ^4^, Paola Rogliani ^5^, Giuseppe Novelli ^2,6,7^, Enrico Garaci ^8^, Guido Rasi ^1^, Paola Sinibaldi-Vallebona ^1,9^, Antonella Minutolo ^1^, Claudia Matteucci ^1^, Emanuela Balestrieri ^1^ and Sandro Grelli ^1,10*^

1. Department of Experimental Medicine, University of Rome Tor Vergata, Rome, Italy
2. Department of Biomedicine and Prevention, University of Rome Tor Vergata, Rome, Italy
3. Martinos Center for Biomedical Imaging and Harvard Medical School, Boston, MA, United States
4. Department of Systems Medicine, University of Rome Tor Vergata, Rome, Italy
5. Respiratory Medicine Unit, Policlinic of Tor Vergata, Rome, Italy
6. Neuromed IRCCS Institute, Pozzilli, IS, Italy
7. University of Nevada, Department of Pharmacology, Reno, USA
8. IRCCS San Raffaele Pisana, Rome, Italy
9. National Research Council, Institute of Translational Pharmacology, Rome, Italy
10. Virology Unit, Policlinic of Tor Vergata, Rome, Italy

(†) These authors contributed equally to this work and share first authorship. Author order was determined on the basis of seniority.

*** Correspondence:** Professor Sandro Grelli, [grelli@med.uniroma2.it](mailto:grelli@med.uniroma2.it)

# Supplementary Tables

**TABLE S1. Primer pair sequences used in the qRT-PCR analysis**

|  |  | **Human primers** | |
| --- | --- | --- | --- |
| **Gene** |  | **Specific Primer Pairs** | |
| #HERV-H | AJ289711 | TTCACTCCATCCTTGGCTAT | CGTCGAGTATCTACGAGCAAT |
| #HERV-K ENV | AF164614 | CATGGCAATTCCCAGTAACTGT | CTCCCTCTTGGGCTCCTTCT |
| #HERV-W ENV | NM_001130925.2 | GTATGTCTGATGGGGGTGGAG | CTAGTCCTTTGTAGGGGCTAGAG |
| #GUSB | NM_000181 | CAGTTCCCTCCAGCTTCAATG | ACCCAGCCGACAAAATGC |
| #IL-1β | NM_000576.2 | CCACCTCCAGGGACAGGATA | AACACGCAGGACAGGTACAG |
| #IL-6 | NM_000600.3 | TGCAATAACCACCCCTGACC | ATTTGCCGAAGAGCCCTCAG |
| #IL-10 | NM_000572.2 | ACATCAAGGCGCATGTGAAC | CACGGCCTTGCTCTTGTTTT |
| #IL-17 | NM_002190.3 | CGGACTGTGATGGTCAACCTGA | GCACTTTGCCTCCCAGATCACA |
| #MCP-1 | NM_002982.4 | AGAATCACCAGCAGCAAGTGTCC | TCCTGAACCCACTTCTGCTTGG |
| #IFN-α | NM_024013.3 | GACTCCATCTTGGCTGTGA | TGATTTCTGCTCTGACAACCT |
| #IFN-β | NM_002176.4 | CTTGGATTCCTACAAAGAAGCAGC | TCCTCCTTCTGGAACTGCTGCA |
| #INF-γ | NM_000619.2 | TCAGCTCTGCATCGTTTTGG | GTTCCATTATCCGCTACATCTGAA |
| #TNF-α | NM_000594.3 | CCCGAGTGACAAGCCTGTAG | TGAGGTACAGGCCCTCTGAT |
| #IL-17RA | NM_001289905.1 | TCATCGTCTGCATGACCTGGAG | GGCTGAGTAGATGATCCAGACC |
| #TLR-3 | NM_003265.3 | GCGCTAAAAAGTGAAGAACTGGAT | GCTGGACATTGTTCAGAAAGAGG |
| #TLR-4 | NM_003266.4 | CCCTGAGGCATTTAGGCAGCTA | AGGTAGAGAGGTGGCTTAGGCT |
| #TLR-7 | NM_016562.4 | AAGCCCTTTCAGAAGTCCAAGTT | GGTGAGCTTGCGGGTTTGT |
| #ACE2 | NM_001371415.1 | TCCATTGGTCTTCTGTCACCCG | AGACCATCCACCTCCACTTCTC |
| **SARS-CoV-2 primer** | | | |
| #SARS-CoV-2  N gene | YP_009724397.2 | AAATTTTGGGGACCAGGAAC | TGGCAGCTGTGTAGGTCAAC |

**Table S2. Median values. interquartile range (IQR) and Kruskall Wallis test and Bonferroni’s correction of HERVs, inflammatory markers and SARS-CoV-2 infection-related genes in SARS-CoV-2 negative positive swab samples**

|  |  |  |  | **HERV-K** | **HERV-W** | **HERV-H** | **IL-1β** | **IL-6** | **IL10** | **IL-17** | **IL-17RA** | **TNF-α** | **MCP-1** | **IFN-α** | **IFN-β** | **IFN-γ** | **TLR-3** | **TLR-4** | **TLR-7** | **ACE2** | **N gene SARS-CoV-2** |
| --- | --- | --- | --- | --- | --- | --- | --- | --- | --- | --- | --- | --- | --- | --- | --- | --- | --- | --- | --- | --- | --- |
| ***Negative swabs*** | | ***Number*** |  | 20 | 20 | 20 | 20 | 20 | 20 | 20 | 20 | 20 | 20 | 20 | 20 | 20 | 20 | 20 | 20 | 20 | 20 |
|  |  | Median |  | 1.810 | 0.900 | 0.130 | 2.055 | 1.405 | 2.955 | 1.925 | 1.155 | 1.550 | 0.650 | 10.210 | 5.020 | 0.035 | 3.415 | 0.945 | 1.180 | 2.470 | 0 |
|  |  | IQR | 25 | 0.522 | 0.247 | 0.025 | 0.545 | 0.457 | 0.370 | 0.060 | 0.212 | 0.892 | 0.192 | 7.480 | 0.330 | 0.006 | 0.650 | 0.507 | 0.417 | 0.472 | 0 |
|  |  |  | 50 | 1.810 | 0.900 | 0.130 | 2.055 | 1.405 | 2.955 | 1.925 | 1.155 | 1.550 | 0.650 | 10.210 | 5.020 | 0.035 | 3,415 | 0,945 | 1,18 | 2,47 | 0 |
|  |  |  | 75 | 3.825 | 4.242 | 0.440 | 3.312 | 2.442 | 11.425 | 12.820 | 13.475 | 3.310 | 2.362 | 12.770 | 14.720 | 0.085 | 8.530 | 2.822 | 3.712 | 13.737 | 0 |
|  |  | ***Number*** |  | 43 | 43 | 43 | 43 | 43 | 43 | 43 | 43 | 43 | 43 | 43 | 43 | 43 | 43 | 43 | 43 | 43 | 43 |
| ***Positive swabs*** | | Median |  | 2280.04 | 259.27 | 0.760 | 65.950 | 3.327.500 | 5.347.880 | 12.730 | 3.390 | 150.550 | 3.380 | 662.960 | 260.880 | 3.190 | 141.330 | 23.240 | 14.330 | 21.810 | 9.750 |
|  |  | IQR | 25 | 1264 | 168.160 | 0.090 | 5.410 | 1.238.100 | 53.450 | 1.950 | 0.270 | 15.300 | 1.310 | 130.560 | 64,72 | 1.270 | 24.510 | 6.240 | 1.920 | 6.070 | 1.770 |
|  |  |  | 50 | 2280.04 | 259.270 | 0.760 | 65.950 | 3.327.500 | 5.347.880 | 12.730 | 3.390 | 150.550 | 3.380 | 662.960 | 260,8 | 3.190 | 141.330 | 23.240 | 14.330 | 21.810 | 9.750 |
|  |  |  | 75 | 6351.6 | 714.740 | 1.720 | 225.050 | 8.554.500 | 16.760.180 | 52.300 | 10.130 | 799.650 | 47.690 | 2.444.130 | 746,41 | 10.720 | 696.010 | 128.630 | 51.030 | 92.640 | 108.300 |
|  |  | ***Number*** |  | 29 | 29 | 29 | 29 | 29 | 29 | 29 | 29 | 29 | 29 | 29 | 29 | 29 | 29 | 29 | 29 | 29 | 29 |
|  | ***Not-HOSP*** | Median |  | 1724.500 | 241.020 | 0.160 | 8.150 | 2622.8 | 11417.430 | 5.480 | 2.260 | 51.020 | 2.270 | 1538.830 | 476.740 | 2.100 | 116.360 | 51.230 | 5.280 | 17.090 | 5.260 |
|  |  | IQR | 25 | 542.230 | 119.115 | 0.050 | 1.830 | 836.3 | 4505.680 | 1.470 | 0.150 | 7.275 | 0.865 | 656.505 | 232.465 | 1.255 | 31.150 | 13.440 | 1.860 | 4.010 | 1.380 |
|  |  |  | 50 | 1724.500 | 241.020 | 0.160 | 8.150 | 2622.8 | 11417.430 | 5.480 | 2.260 | 51.020 | 2.270 | 1.538.830 | 476.740 | 2.100 | 116.360 | 51.230 | 5.280 | 17.090 | 5.260 |
|  |  |  | 75 | 3550.700 | 403.005 | 0.890 | 129.075 | 5224.500 | 22378.190 | 13.265 | 9.065 | 188.780 | 6.040 | 3560.710 | 1.095.075 | 8.985 | 321.125 | 195.795 | 27.215 | 56.680 | 9.835 |
|  |  | ***Number*** |  | 14 | 14 | 14 | 14 | 14 | 14 | 14 | 14 | 14 | 14 | 14 | 14 | 14 | 14 | 14 | 14 | 14 | 14 |
|  | ***HOSP*** | Median |  | 11721.950 | 694.115 | 2.250 | 165,915 | 15063.5 | 14.800 | 87.335 | 6.405 | 1258.150 | 49.485 | 89.190 | 27.650 | 7.310 | 679.580 | 3.930 | 114.660 | 63.925 | 331.140 |
|  |  | IQR | 25 | 3147.400 | 340.192 | 1.650 | 56.445 | 4423.775 | 2.987 | 23.770 | 1.520 | 486,817 | 14.757 | 13.212 | 8.640 | 2.725 | 15.582 | 0.375 | 12.355 | 12.080 | 96.525 |
|  |  |  | 50 | 11721.950 | 694.115 | 2.250 | 165.915 | 15063.5 | 14.800 | 87.335 | 6.405 | 1258.150 | 49.485 | 89.190 | 27.650 | 7.310 | 679.580 | 3.930 | 114.660 | 63.925 | 331.140 |
|  |  |  | 75 | 22363.500 | 1026.425 | 7.380 | 369.927 | 57409.2 | 65.207 | 198.330 | 19.620 | 2112.350 | 128.740 | 140.402 | 75.575 | 19.487 | 3594.175 | 33.127 | 201.737 | 282.172 | 1570.127 |
| ***Negative vs Positive Not-HOSP*** | | |  | <0.001 | <0.001 | 0.226 | 0.011 | <0.001 | <0.001 | 0.455 | 0.288 | 0.003 | 0.197 | <0.001 | <0.001 | <0.001 | <0.001 | <0.001 | 0.030 | 0.035 | <0.001 |
| ***Negative vs Positive HOSP*** | | |  | <0.001 | <0.001 | 0.002 | <0.001 | <0.001 | 0.650 | <0.001 | 0.288 | <0.001 | <0.001 | 0.254 | 0.267 | <0.001 | <0.001 | 0.377 | <0.001 | <0.001 | <0.001 |
| ***Not-HOSP vs HOSP*** | |  |  | 0.024 | 0.262 | 0.001 | 0.031 | 0.045 | <0.001 | 0.001 | 0.288 | 0.001 | 0.001 | <0.001 | <0.001 | 1 | 1 | 0.004 | 0.040 | 0.186 | 0.002 |

**Supplemental Table 3. Median values. interquartile range (IQR) and Mann Whitney test of HERVs, inflammatory markers and SARS-CoV-2 infection-related genes in SARS-CoV-2 positive swabs samples, stratified by oxygen needs of COVID-19 patients**

|  |  |  |  | | **HERV-K** | **HERV-W** | **HERV-H** | **IL-1β** | **IL-6** | **IL-10** | **IL-17** | **IL-17RA** | **TNF-α** | **MCP-1** | **IFNα** | **IFN-β** | **IFN-γ** | **TLR-3** | **TLR-4** | **TLR-7** | **ACE2** | **N gene SARS-CoV-2** |
| --- | --- | --- | --- | --- | --- | --- | --- | --- | --- | --- | --- | --- | --- | --- | --- | --- | --- | --- | --- | --- | --- | --- |
| ***Positive swabs*** | | ***Number*** | |  | 6 | 6 | 6 | 6 | 6 | 6 | 6 | 6 | 6 | 6 | 6 | 6 | 6 | 6 | 6 | 6 | 6 | 6 |
|  | ***No oxygen supported*** | Median | |  | 8054.75 | 302.145 | 5.4632 | 46.94 | 4058.35 | 22.75 | 112.43 | 7.145 | 1633 | 47.89 | 11.875 | 7.94 | 3.815 | 420.905 | 4.265 | 114.660 | 77.650 | 285.025 |
|  |  | IQR | | 25 | 3147.4 | 134.142 | 1.8501 | 9.572 | 1684.73 | 2.39 | 18.487 | 4.515 | 649.172 | 17 | 4.955 | 4.117 | 0.480 | 96.337 | 1.1325 | 10.750 | 12.080 | 83.537 |
|  |  |  | | 50 | 8054.75 | 302.145 | 5.4632 | 46.94 | 4058.35 | 22.75 | 112.43 | 7.145 | 1633 | 47.89 | 11.875 | 7.94 | 3.815 | 420.905 | 4.265 | 114.660 | 77.650 | 285.025 |
|  |  |  | | 75 | 47745 | 441.152 | 17.09 | 87.862 | 7227.175 | 65.652 | 306.137 | 61.35 | 3707.425 | 168.79 | 31.505 | 10.85 | 30.400 | 45247.300 | 6.975 | 926.912 | 17153.377 | 539.297 |
|  |  | ***Number*** | |  | 8 | 8 | 8 | 8 | 8 | 8 | 8 | 8 | 8 | 8 | 8 | 8 | 8 | 8 | 8 | 8 | 8 | 8 |
|  | ***Oxygen supported*** | Median | |  | 15635 | 1020.4 | 1.858 | 347.185 | 54152.5 | 7.47 | 59.405 | 3.985 | 991.04 | 57.73 | 128.935 | 68.955 | 8.460 | 976.125 | 3.930 | 84.995 | 46.455 | 1353.995 |
|  |  | IQR | | 25 | 2598.45 | 760.382 | 1.609 | 193.815 | 18398.75 | 3.242 | 27.582 | 1.46 | 380.072 | 14.232 | 109.155 | 43.63 | 4.602 | 1.475 | 0.130 | 8.910 | 10.172 | 83.572 |
|  |  |  | | 50 | 15635 | 1020.4 | 1.858 | 347.185 | 54152.5 | 7.47 | 59.405 | 3.985 | 991.04 | 57.73 | 128.935 | 68.955 | 8.460 | 976.125 | 3.930 | 84.995 | 46.455 | 1353.995 |
|  |  |  | | 75 | 21552 | 1176.425 | 3.356 | 516.65 | 86051 | 88.722 | 169.252 | 13.9 | 1712.1 | 115.515 | 183.295 | 102.297 | 20.417 | 6157.925 | 274.080 | 170.175 | 195.050 | 2014.217 |
| ***No oxygen supported vs oxygen supported*** | |  |  | | 0.142 | **0.003** | 0.954 | **0.001** | **0.002** | 0.950 | 0.512 | 0.751 | 1.000 | 0.850 | **0.003** | **0.002** | 0.850 | 0.662 | 0.228 | 0.178 | 0.246 | 0.416 |

**Supplemental Table 4. Median values and interquartile range (IQR) of HERVs and inflammatory markers in FaDu cells treated in vitro with Spike protein**

|  |  |  |  | **HERV-K** | **HERV-W** | **HERV-H** | **IL-1β** | **IL-6** | **IL-10** | **IL-17** | **TNF-α** | **MCP-1** | **IFNα** | **IFN-β** | **IFN-γ** | **TLR-3** | **TLR-4** | **TLR-7** | **ACE2** |
| --- | --- | --- | --- | --- | --- | --- | --- | --- | --- | --- | --- | --- | --- | --- | --- | --- | --- | --- | --- |
| ***Fadu cells untreated (3h)*** | | ***Number*** |  | 9 | 9 | 9 | 9 | 9 | 9 | 9 | 9 | 9 | 9 | 9 | 9 | 9 | 9 | 9 | 9 |
|  |  | Median |  | 1.2226 | .8942 | 3.9367 | .9865 | 1.1754 | 1.0824 | 1.6935 | 1.0615 | 1.0249 | .8258 | .9510 | .9474 | 1.3341 | 1.0740 | .8934 | .8137 |
|  |  | IQR | 25 | .4476 | .6749 | 0.6083 | .7041 | .3857 | .8349 | .3934 | .7946 | .8980 | .6589 | .7170 | .6068 | .5342 | .5575 | .7640 | .6918 |
|  |  |  | 50 | 1.2226 | .8942 | 3.9367 | .9865 | 1.1754 | 1.0824 | 1.6935 | 1.0615 | 1.0249 | .8258 | .9510 | .9474 | 1.3341 | 1.0740 | .8934 | .8137 |
|  |  |  | 75 | 1.8440 | 1.6566 | 7.4676 | 1.4003 | 2.2134 | 1.1196 | 1.7736 | 1.1719 | 1.0938 | 1.9000 | 1.4950 | 1.6131 | 1.4536 | 1.8210 | 1.4163 | 2.0109 |
|  |  | ***Number*** |  | 9 | 9 | 9 | 9 | 9 | 9 | 9 | 9 | 9 | 9 | 9 | 9 | 9 | 9 | 9 | 9 |
| ***Fadu cells untreated (8h)*** | | Median |  | 1.6461 | 1.1725 | 1.591 | .5655 | 1.4308 | 1.5002 | .8563 | 1.1600 | 1.6386 | 12.5827 | 4.3926 | 2.6666 | 3.2907 | 9.5256 | 2.4552 | 3.9660 |
|  |  | IQR | 25 | .4236 | .8704 | 0.9879 | .3773 | .9332 | .7320 | .2705 | .6729 | 1.2926 | 7.4114 | 1.3143 | 1.8828 | 1.6409 | 6.9490 | 2.0443 | 2.7657 |
|  |  |  | 50 | 1.6461 | 1.1725 | 1.591 | .5655 | 1.4308 | 1.5002 | .8563 | 1.1600 | 1.6386 | 12.5827 | 4.3926 | 2.6666 | 3.2907 | 9.5256 | 2.4552 | 3.9660 |
|  |  |  | 75 | 2.6075 | 1.7830 | 14.9123 | 1.3362 | 2.2172 | 1.8417 | 1.3742 | 1.6780 | 2.8987 | 20.4405 | 6.5057 | 6.9949 | 7.9072 | 18.7639 | 4.3370 | 4.7790 |
|  |  | ***Number*** |  | 9 | 9 | 9 | 9 | 9 | 9 | 9 | 9 | 9 | 9 | 9 | 9 | 9 | 9 | 9 | 9 |
| ***Fadu cells untreated (24h)*** | | Median |  | 1.2473 | 1.3318 | 2.2883 | .9825 | 2.6946 | 1.0680 | 2.3702 | 2.0186 | .7200 | 3.6701 | 18.7331 | 3.0326 | 2.7911 | 4.1171 | 1.4903 | 1.8286 |
|  |  | IQR | 25 | 1.0830 | 1.2256 | 1.2206 | .6853 | .8351 | .9125 | 1.0067 | 1.4882 | .6456 | 2.3817 | 17.0628 | 1.4784 | 1.8231 | 2.5206 | .8139 | .7029 |
|  |  |  | 50 | 1.2473 | 1.3318 | 2.2883 | .9825 | 2.6946 | 1.0680 | 2.3702 | 2.0186 | .7200 | 3.6701 | 18.7331 | 3.0326 | 2.7911 | 4.1171 | 1.4903 | 1.8286 |
|  |  |  | 75 | 4.2770 | 4.0332 | 4.792 | 1.7983 | 3.5913 | 1.2012 | 3.2544 | 2.2919 | 1.5158 | 5.0361 | 21.2907 | 3.3572 | 4.3105 | 8.3250 | 4.6800 | 2.1525 |
|  |  | ***Number*** |  | 9 | 9 | 9 | 9 | 9 | 9 | 9 | 9 | 9 | 9 | 9 | 9 | 9 | 9 | 9 | 9 |
| ***Fadu cells tr Spike protein (3h)*** | | Median |  | 24.5699 | 26.9558 | 3.8823 | 15.3746 | 43.2635 | 35.4245 | 8.0975 | 145.3446 | 4.6785 | 3.0938 | 2.8030 | 2.7039 | 8.3407 | 16.3660 | 17.9724 | 5.9364 |
|  |  | IQR | 25 | 17.6649 | 21.0536 | 1.5932 | 10.1416 | 14.2489 | 22.4868 | 5.7793 | 120.3419 | 4.2031 | 2.2273 | 1.4071 | 1.7040 | 4.8847 | 6.1015 | 8.1550 | 5.0908 |
|  |  |  | 50 | 24.5699 | 26.9558 | 3.8823 | 15.3746 | 43.2635 | 35.4245 | 8.0975 | 145.3446 | 4.6785 | 3.0938 | 2.8030 | 2.7039 | 8.3407 | 16.3660 | 17.9724 | 5.9364 |
|  |  |  | 75 | 52.2848 | 33.5498 | 7.8161 | 24.4626 | 48.3785 | 49.4814 | 10.2518 | 168.5671 | 10.5318 | 5.2519 | 3.5863 | 6.3418 | 12.3188 | 19.9000 | 25.6656 | 7.0873 |
|  |  | ***Number*** |  | 9 | 9 | 9 | 9 | 9 | 9 | 9 | 9 | 9 | 9 | 9 | 9 | 9 | 9 | 9 | 9 |
| ***Fadu cells tr Spike protein (8h)*** | | Median |  | 35.7414 | 68.4948 | 3.5796 | 13.4556 | 51.4610 | 71.6320 | 53.3551 | 73.2411 | 5.2095 | 8.0324 | 16.3930 | 11.3372 | 9.5687 | 32.7738 | 11.2095 | 7.4428 |
|  |  | IQR | 25 | 30.4931 | 53.1916 | 0.0006 | 12.6902 | 45.5071 | 53.0856 | 46.1270 | 43.2562 | 4.1615 | 5.0837 | 13.5379 | 10.0659 | 8.2727 | 24.3781 | 8.4891 | 6.4157 |
|  |  |  | 50 | 35.7414 | 68.4948 | 3.5796 | 13.4556 | 51.4610 | 71.6320 | 53.3551 | 73.2411 | 5.2095 | 8.0324 | 16.3930 | 11.3372 | 9.5687 | 32.7738 | 11.2095 | 7.4428 |
|  |  |  | 75 | 47.9744 | 80.5659 | 5.3465 | 15.1108 | 116.8379 | 123.3904 | 63.2867 | 117.7849 | 6.7584 | 8.9438 | 20.0278 | 12.8061 | 19.5536 | 42.4058 | 13.1509 | 8.4489 |
|  |  | ***Number*** |  | 9 | 9 | 9 | 9 | 9 | 9 | 9 | 9 | 9 | 9 | 9 | 9 | 9 | 9 | 9 | 9 |
| ***Fadu cells tr Spike protein (24h)*** | | Median |  | 8.6129 | 19.2184 | 0.8543 | 2.9928 | 5.0281 | 1.2636 | 8.8328 | 11.9864 | 1.1858 | 13.3611 | 51.1798 | 2.2236 | 26.5205 | 15.5305 | 18.0913 | 6.2121 |
|  |  | IQR | 25 | 7.1299 | 16.2208 | 0.3113 | 1.7870 | 3.9833 | .9384 | 3.9663 | 8.7933 | 1.0097 | 9.7061 | 41.3248 | 1.7373 | 11.4990 | 12.1873 | 13.3207 | 2.5991 |
|  |  |  | 50 | 8.6129 | 19.2184 | 0.8543 | 2.9928 | 5.0281 | 1.2636 | 8.8328 | 11.9864 | 1.1858 | 13.3611 | 51.1798 | 2.2236 | 26.5205 | 15.5305 | 18.0913 | 6.2121 |
|  |  |  | 75 | 13.5326 | 23.4231 | 1.6485 | 3.3087 | 9.8332 | 4.6770 | 9.5389 | 13.9130 | 1.2726 | 17.2570 | 63.0236 | 3.1671 | 55.4502 | 21.9650 | 21.0697 | 11.6195 |

**Supplemental Table 5. Results of Kruskall Wallis test in FaDu cells treated in vitro with Spike protein**

| **Dependent Variable** | **(I) V2** | **(J) V2** | **Mean Difference (I-J)** | **Std. Error** | **Sig.** | **95% Confidence Interval** | |
| --- | --- | --- | --- | --- | --- | --- | --- |
|  |  |  |  |  |  | ***Lower Bound*** | ***Upper Bound*** |
| **HERV-W** | **Untreated cells 3h** | **Spike 3h** | -35.759325700000* | 4.54135637 | 0 | -50.24082961 | -21.27782179 |
|  |  | **Untreated cells 8h** | -0.188327194 | 4.54135637 | 1 | -14.6698311 | 14.29317672 |
|  |  | **Spike 8h** | -65.835518740000* | 4.54135637 | 0 | -80.31702265 | -51.35401483 |
|  |  | **Untreated cells 24h** | -1.217637677 | 4.54135637 | 1 | -15.69914159 | 13.26386623 |
|  |  | **Spike 24h** | -18.398159510000* | 4.54135637 | 0.005 | -32.87966342 | -3.916655597 |
|  | **Spike 3h** | **Untreated cells 3h** | 35.759325700000* | 4.54135637 | 0 | 21.27782179 | 50.24082961 |
|  |  | **Untreated cells 8h** | 35.570998500000* | 4.54135637 | 0 | 21.08949459 | 50.05250242 |
|  |  | **Spike 8h** | -30.076193040000* | 4.54135637 | 0 | -44.55769695 | -15.59468913 |
|  |  | **Untreated cells 24h** | 34.541688020000* | 4.54135637 | 0 | 20.06018411 | 49.02319193 |
|  |  | **Spike 24h** | 17.361166190000* | 4.54135637 | 0.009 | 2.879662279 | 31.8426701 |
|  | **Untreated cells 8h** | **Untreated cells 3h** | 0.188327194 | 4.54135637 | 1 | -14.29317672 | 14.6698311 |
|  |  | **Spike 3h** | -35.570998500000* | 4.54135637 | 0 | -50.05250242 | -21.08949459 |
|  |  | **Spike 8h** | -65.647191540000* | 4.54135637 | 0 | -80.12869546 | -51.16568763 |
|  |  | **Untreated cells 24h** | -1.029310484 | 4.54135637 | 1 | -15.51081439 | 13.45219343 |
|  |  | **Spike 24h** | -18.209832310000* | 4.54135637 | 0.006 | -32.69133623 | -3.728328404 |
|  | **Spike 8h** | **Untreated cells 3h** | 65.835518740000* | 4.54135637 | 0 | 51.35401483 | 80.31702265 |
|  |  | **Spike 3h** | 30.076193040000* | 4.54135637 | 0 | 15.59468913 | 44.55769695 |
|  |  | **Untreated cells 8h** | 65.647191540000* | 4.54135637 | 0 | 51.16568763 | 80.12869546 |
|  |  | **Untreated cells 24h** | 64.617881060000* | 4.54135637 | 0 | 50.13637715 | 79.09938497 |
|  |  | **Spike 24h** | 47.437359230000* | 4.54135637 | 0 | 32.95585532 | 61.91886314 |
|  | **Untreated cells 24h** | **Untreated cells 3h** | 1.217637677 | 4.54135637 | 1 | -13.26386623 | 15.69914159 |
|  |  | **Spike 3h** | -34.541688020000* | 4.54135637 | 0 | -49.02319193 | -20.06018411 |
|  |  | **Untreated cells 8h** | 1.029310484 | 4.54135637 | 1 | -13.45219343 | 15.51081439 |
|  |  | **Spike 8h** | -64.617881060000* | 4.54135637 | 0 | -79.09938497 | -50.13637715 |
|  |  | **Spike 24h** | -17.180521830000* | 4.54135637 | 0.01 | -31.66202574 | -2.69901792 |
|  | **Spike 24h** | **Untreated cells 3h** | 18.398159510000* | 4.54135637 | 0.005 | 3.916655597 | 32.87966342 |
|  |  | **Spike 3h** | -17.361166190000* | 4.54135637 | 0.009 | -31.8426701 | -2.879662279 |
|  |  | **Untreated cells 8h** | 18.209832310000* | 4.54135637 | 0.006 | 3.728328404 | 32.69133623 |
|  |  | **Spike 8h** | -47.437359230000* | 4.54135637 | 0 | -61.91886314 | -32.95585532 |
|  |  | **Untreated cells 24h** | 17.180521830000* | 4.54135637 | 0.01 | 2.69901792 | 31.66202574 |
| **HERV-K** | **Untreated cells 3h** | **Spike 3h** | -34.326296550000* | 4.2651514 | 0 | -47.92703644 | -20.72555666 |
|  |  | **Untreated cells 8h** | 0.312977879 | 4.2651514 | 1 | -13.28776201 | 13.91371777 |
|  |  | **Spike 8h** | -38.174184780000* | 4.2651514 | 0 | -51.77492467 | -24.57344489 |
|  |  | **Untreated cells 24h** | -1.185405497 | 4.2651514 | 1 | -14.78614538 | 12.41533439 |
|  |  | **Spike 24h** | -9.032099008 | 4.2651514 | 0.639 | -22.6328389 | 4.568640878 |
|  | **Spike 3h** | **Untreated cells 3h** | 34.326296550000* | 4.2651514 | 0 | 20.72555666 | 47.92703644 |
|  |  | **Untreated cells 8h** | 34.639274430000* | 4.2651514 | 0 | 21.03853454 | 48.24001432 |
|  |  | **Spike 8h** | -3.84788823 | 4.2651514 | 1 | -17.44862812 | 9.752851657 |
|  |  | **Untreated cells 24h** | 33.140891050000* | 4.2651514 | 0 | 19.54015117 | 46.74163094 |
|  |  | **Spike 24h** | 25.294197540000* | 4.2651514 | 0 | 11.69345765 | 38.89493743 |
|  | **Untreated cells 8h** | **Untreated cells 3h** | -0.312977879 | 4.2651514 | 1 | -13.91371777 | 13.28776201 |
|  |  | **Spike 3h** | -34.639274430000* | 4.2651514 | 0 | -48.24001432 | -21.03853454 |
|  |  | **Spike 8h** | -38.487162660000* | 4.2651514 | 0 | -52.08790255 | -24.88642277 |
|  |  | **Untreated cells 24h** | -1.498383376 | 4.2651514 | 1 | -15.09912326 | 12.10235651 |
|  |  | **Spike 24h** | -9.345076887 | 4.2651514 | 0.545 | -22.94581677 | 4.255662999 |
|  | **Spike 8h** | **Untreated cells 3h** | 38.174184780000* | 4.2651514 | 0 | 24.57344489 | 51.77492467 |
|  |  | **Spike 3h** | 3.84788823 | 4.2651514 | 1 | -9.752851657 | 17.44862812 |
|  |  | **Untreated cells 8h** | 38.487162660000* | 4.2651514 | 0 | 24.88642277 | 52.08790255 |
|  |  | **Untreated cells 24h** | 36.988779280000* | 4.2651514 | 0 | 23.3880394 | 50.58951917 |
|  |  | **Spike 24h** | 29.142085770000* | 4.2651514 | 0 | 15.54134588 | 42.74282566 |
|  | **Untreated cells 24h** | **Untreated cells 3h** | 1.185405497 | 4.2651514 | 1 | -12.41533439 | 14.78614538 |
|  |  | **Spike 3h** | -33.140891050000* | 4.2651514 | 0 | -46.74163094 | -19.54015117 |
|  |  | **Untreated cells 8h** | 1.498383376 | 4.2651514 | 1 | -12.10235651 | 15.09912326 |
|  |  | **Spike 8h** | -36.988779280000* | 4.2651514 | 0 | -50.58951917 | -23.3880394 |
|  |  | **Spike 24h** | -7.846693511 | 4.2651514 | 1 | -21.4474334 | 5.754046375 |
|  | **Spike 24h** | **Untreated cells 3h** | 9.032099008 | 4.2651514 | 0.639 | -4.568640878 | 22.6328389 |
|  |  | **Spike 3h** | -25.294197540000* | 4.2651514 | 0 | -38.89493743 | -11.69345765 |
|  |  | **Untreated cells 8h** | 9.345076887 | 4.2651514 | 0.545 | -4.255662999 | 22.94581677 |
|  |  | **Spike 8h** | -29.142085770000* | 4.2651514 | 0 | -42.74282566 | -15.54134588 |
|  |  | **Untreated cells 24h** | 7.846693511 | 4.2651514 | 1 | -5.754046375 | 21.4474334 |
| **HERV-H** | **Untreated cells 3h** | **Spike 3h** | -0.535183393 | 2.37476968 | 1 | -8.10786291 | 7.037496125 |
|  |  | **Untreated cells 8h** | -2.129902158 | 2.37476968 | 1 | -9.702581675 | 5.44277736 |
|  |  | **Spike 8h** | 0.495279303 | 2.37476968 | 1 | -7.077400215 | 8.06795882 |
|  |  | **Untreated cells 24h** | 1.20288828 | 2.37476968 | 1 | -6.369791237 | 8.775567798 |
|  |  | **Spike 24h** | 2.955916868 | 2.37476968 | 1 | -4.61676265 | 10.52859639 |
|  | **Spike 3h** | **Untreated cells 3h** | 0.535183393 | 2.37476968 | 1 | -7.037496125 | 8.10786291 |
|  |  | **Untreated cells 8h** | -1.594718765 | 2.37476968 | 1 | -9.167398283 | 5.977960753 |
|  |  | **Spike 8h** | 1.030462695 | 2.37476968 | 1 | -6.542216822 | 8.603142213 |
|  |  | **Untreated cells 24h** | 1.738071673 | 2.37476968 | 1 | -5.834607845 | 9.310751191 |
|  |  | **Spike 24h** | 3.49110026 | 2.37476968 | 1 | -4.081579257 | 11.06377978 |
|  | **Untreated cells 8h** | **Untreated cells 3h** | 2.129902158 | 2.37476968 | 1 | -5.44277736 | 9.702581675 |
|  |  | **Spike 3h** | 1.594718765 | 2.37476968 | 1 | -5.977960753 | 9.167398283 |
|  |  | **Spike 8h** | 2.62518146 | 2.37476968 | 1 | -4.947498057 | 10.19786098 |
|  |  | **Untreated cells 24h** | 3.332790438 | 2.37476968 | 1 | -4.23988908 | 10.90546996 |
|  |  | **Spike 24h** | 5.085819025 | 2.37476968 | 0.607 | -2.486860492 | 12.65849854 |
|  | **Spike 8h** | **Untreated cells 3h** | -0.495279303 | 2.37476968 | 1 | -8.06795882 | 7.077400215 |
|  |  | **Spike 3h** | -1.030462695 | 2.37476968 | 1 | -8.603142213 | 6.542216822 |
|  |  | **Untreated cells 8h** | -2.62518146 | 2.37476968 | 1 | -10.19786098 | 4.947498057 |
|  |  | **Untreated cells 24h** | 0.707608978 | 2.37476968 | 1 | -6.86507054 | 8.280288495 |
|  |  | **Spike 24h** | 2.460637565 | 2.37476968 | 1 | -5.112041953 | 10.03331708 |
|  | **Untreated cells 24h** | **Untreated cells 3h** | -1.20288828 | 2.37476968 | 1 | -8.775567798 | 6.369791237 |
|  |  | **Spike 3h** | -1.738071673 | 2.37476968 | 1 | -9.310751191 | 5.834607845 |
|  |  | **Untreated cells 8h** | -3.332790438 | 2.37476968 | 1 | -10.90546996 | 4.23988908 |
|  |  | **Spike 8h** | -0.707608978 | 2.37476968 | 1 | -8.280288495 | 6.86507054 |
|  |  | **Spike 24h** | 1.753028587 | 2.37476968 | 1 | -5.81965093 | 9.325708105 |
|  | **Spike 24h** | **Untreated cells 3h** | -2.955916868 | 2.37476968 | 1 | -10.52859639 | 4.61676265 |
|  |  | **Spike 3h** | -3.49110026 | 2.37476968 | 1 | -11.06377978 | 4.081579257 |
|  |  | **Untreated cells 8h** | -5.085819025 | 2.37476968 | 0.607 | -12.65849854 | 2.486860492 |
|  |  | **Spike 8h** | -2.460637565 | 2.37476968 | 1 | -10.03331708 | 5.112041953 |
|  |  | **Untreated cells 24h** | -1.753028587 | 2.37476968 | 1 | -9.325708105 | 5.81965093 |
| **IL-1β** | **Untreated cells 3h** | **Spike 3h** | -0.241387955 | 0.40912826 | 1 | -1.546018568 | 1.063242658 |
|  |  | **Untreated cells 8h** | 0.275342226 | 0.40912826 | 1 | -1.029288387 | 1.579972838 |
|  |  | **Spike 8h** | -12.634548160000* | 0.40912826 | 0 | -13.93917878 | -11.32991755 |
|  |  | **Untreated cells 24h** | -0.157277436 | 0.40912826 | 1 | -1.461908048 | 1.147353177 |
|  |  | **Spike 24h** | -1.641697867000* | 0.40912826 | 0.006 | -2.946328479 | -0.337067254 |
|  | **Spike 3h** | **Untreated cells 3h** | 0.241387955 | 0.40912826 | 1 | -1.063242658 | 1.546018568 |
|  |  | **Untreated cells 8h** | 0.516730181 | 0.40912826 | 1 | -0.787900432 | 1.821360793 |
|  |  | **Spike 8h** | -12.393160210000* | 0.40912826 | 0 | -13.69779082 | -11.0885296 |
|  |  | **Untreated cells 24h** | 0.084110519 | 0.40912826 | 1 | -1.220520093 | 1.388741132 |
|  |  | **Spike 24h** | -1.400309912000* | 0.40912826 | 0.027 | -2.704940524 | -0.095679299 |
|  | **Untreated cells 8h** | **Untreated cells 3h** | -0.275342226 | 0.40912826 | 1 | -1.579972838 | 1.029288387 |
|  |  | **Spike 3h** | -0.516730181 | 0.40912826 | 1 | -1.821360793 | 0.787900432 |
|  |  | **Spike 8h** | -12.909890390000* | 0.40912826 | 0 | -14.214521 | -11.60525978 |
|  |  | **Untreated cells 24h** | -0.432619662 | 0.40912826 | 1 | -1.737250274 | 0.872010951 |
|  |  | **Spike 24h** | -1.917040093000* | 0.40912826 | 0.001 | -3.221670705 | -0.61240948 |
|  | **Spike 8h** | **Untreated cells 3h** | 12.634548160000* | 0.40912826 | 0 | 11.32991755 | 13.93917878 |
|  |  | **Spike 3h** | 12.393160210000* | 0.40912826 | 0 | 11.0885296 | 13.69779082 |
|  |  | **Untreated cells 8h** | 12.909890390000* | 0.40912826 | 0 | 11.60525978 | 14.214521 |
|  |  | **Untreated cells 24h** | 12.477270730000* | 0.40912826 | 0 | 11.17264012 | 13.78190134 |
|  |  | **Spike 24h** | 10.992850300000* | 0.40912826 | 0 | 9.688219684 | 12.29748091 |
|  | **Untreated cells 24h** | **Untreated cells 3h** | 0.157277436 | 0.40912826 | 1 | -1.147353177 | 1.461908048 |
|  |  | **Spike 3h** | -0.084110519 | 0.40912826 | 1 | -1.388741132 | 1.220520093 |
|  |  | **Untreated cells 8h** | 0.432619662 | 0.40912826 | 1 | -0.872010951 | 1.737250274 |
|  |  | **Spike 8h** | -12.477270730000* | 0.40912826 | 0 | -13.78190134 | -11.17264012 |
|  |  | **Spike 24h** | -1.484420431000* | 0.40912826 | 0.016 | -2.789051044 | -0.179789818 |
|  | **Spike 24h** | **Untreated cells 3h** | 1.641697867000* | 0.40912826 | 0.006 | 0.337067254 | 2.946328479 |
|  |  | **Spike 3h** | 1.400309912000* | 0.40912826 | 0.027 | 0.095679299 | 2.704940524 |
|  |  | **Untreated cells 8h** | 1.917040093000* | 0.40912826 | 0.001 | 0.61240948 | 3.221670705 |
|  |  | **Spike 8h** | -10.992850300000* | 0.40912826 | 0 | -12.29748091 | -9.688219684 |
|  |  | **Untreated cells 24h** | 1.484420431000* | 0.40912826 | 0.016 | 0.179789818 | 2.789051044 |
| **IL-6** | **Untreated cells 3h** | **Spike 3h** | -6.050356991 | 10.5776938 | 1 | -39.78056918 | 27.6798552 |
|  |  | **Untreated cells 8h** | -0.298452819 | 10.5776938 | 1 | -34.02866501 | 33.43175938 |
|  |  | **Spike 8h** | -73.810318250000* | 10.5776938 | 0 | -107.5405304 | -40.08010606 |
|  |  | **Untreated cells 24h** | -1.155674379 | 10.5776938 | 1 | -34.88588657 | 32.57453781 |
|  |  | **Spike 24h** | -4.954254596 | 10.5776938 | 1 | -38.68446679 | 28.7759576 |
|  | **Spike 3h** | **Untreated cells 3h** | 6.050356991 | 10.5776938 | 1 | -27.6798552 | 39.78056918 |
|  |  | **Untreated cells 8h** | 5.751904172 | 10.5776938 | 1 | -27.97830802 | 39.48211637 |
|  |  | **Spike 8h** | -67.759961260000* | 10.5776938 | 0 | -101.4901735 | -34.02974906 |
|  |  | **Untreated cells 24h** | 4.894682611 | 10.5776938 | 1 | -28.83552958 | 38.62489481 |
|  |  | **Spike 24h** | 1.096102394 | 10.5776938 | 1 | -32.6341098 | 34.82631459 |
|  | **Untreated cells 8h** | **Untreated cells 3h** | 0.298452819 | 10.5776938 | 1 | -33.43175938 | 34.02866501 |
|  |  | **Spike 3h** | -5.751904172 | 10.5776938 | 1 | -39.48211637 | 27.97830802 |
|  |  | **Spike 8h** | -73.511865430000* | 10.5776938 | 0 | -107.2420776 | -39.78165324 |
|  |  | **Untreated cells 24h** | -0.85722156 | 10.5776938 | 1 | -34.58743375 | 32.87299063 |
|  |  | **Spike 24h** | -4.655801778 | 10.5776938 | 1 | -38.38601397 | 29.07441042 |
|  | **Spike 8h** | **Untreated cells 3h** | 73.810318250000* | 10.5776938 | 0 | 40.08010606 | 107.5405304 |
|  |  | **Spike 3h** | 67.759961260000* | 10.5776938 | 0 | 34.02974906 | 101.4901735 |
|  |  | **Untreated cells 8h** | 73.511865430000* | 10.5776938 | 0 | 39.78165324 | 107.2420776 |
|  |  | **Untreated cells 24h** | 72.654643870000* | 10.5776938 | 0 | 38.92443168 | 106.3848561 |
|  |  | **Spike 24h** | 68.856063650000* | 10.5776938 | 0 | 35.12585146 | 102.5862758 |
|  | **Untreated cells 24h** | **Untreated cells 3h** | 1.155674379 | 10.5776938 | 1 | -32.57453781 | 34.88588657 |
|  |  | **Spike 3h** | -4.894682611 | 10.5776938 | 1 | -38.62489481 | 28.83552958 |
|  |  | **Untreated cells 8h** | 0.85722156 | 10.5776938 | 1 | -32.87299063 | 34.58743375 |
|  |  | **Spike 8h** | -72.654643870000* | 10.5776938 | 0 | -106.3848561 | -38.92443168 |
|  |  | **Spike 24h** | -3.798580217 | 10.5776938 | 1 | -37.52879241 | 29.93163198 |
|  | **Spike 24h** | **Untreated cells 3h** | 4.954254596 | 10.5776938 | 1 | -28.7759576 | 38.68446679 |
|  |  | **Spike 3h** | -1.096102394 | 10.5776938 | 1 | -34.82631459 | 32.6341098 |
|  |  | **Untreated cells 8h** | 4.655801778 | 10.5776938 | 1 | -29.07441042 | 38.38601397 |
|  |  | **Spike 8h** | -68.856063650000* | 10.5776938 | 0 | -102.5862758 | -35.12585146 |
|  |  | **Untreated cells 24h** | 3.798580217 | 10.5776938 | 1 | -29.93163198 | 37.52879241 |
| **IL-10** | **Untreated cells 3h** | **Spike 3h** | -12.0489996 | 8.20119121 | 1 | -38.20100639 | 14.10300719 |
|  |  | **Untreated cells 8h** | -0.335217796 | 8.20119121 | 1 | -26.48722459 | 25.81678899 |
|  |  | **Spike 8h** | -81.881503500000* | 8.20119121 | 0 | -108.0335103 | -55.72949671 |
|  |  | **Untreated cells 24h** | -0.052553267 | 8.20119121 | 1 | -26.20456006 | 26.09945352 |
|  |  | **Spike 24h** | -1.563049824 | 8.20119121 | 1 | -27.71505661 | 24.58895697 |
|  | **Spike 3h** | **Untreated cells 3h** | 12.0489996 | 8.20119121 | 1 | -14.10300719 | 38.20100639 |
|  |  | **Untreated cells 8h** | 11.71378181 | 8.20119121 | 1 | -14.43822498 | 37.8657886 |
|  |  | **Spike 8h** | -69.832503890000* | 8.20119121 | 0 | -95.98451068 | -43.6804971 |
|  |  | **Untreated cells 24h** | 11.99644634 | 8.20119121 | 1 | -14.15556045 | 38.14845313 |
|  |  | **Spike 24h** | 10.48594978 | 8.20119121 | 1 | -15.66605701 | 36.63795657 |
|  | **Untreated cells 8h** | **Untreated cells 3h** | 0.335217796 | 8.20119121 | 1 | -25.81678899 | 26.48722459 |
|  |  | **Spike 3h** | -11.71378181 | 8.20119121 | 1 | -37.8657886 | 14.43822498 |
|  |  | **Spike 8h** | -81.546285700000* | 8.20119121 | 0 | -107.6982925 | -55.39427891 |
|  |  | **Untreated cells 24h** | 0.282664529 | 8.20119121 | 1 | -25.86934226 | 26.43467132 |
|  |  | **Spike 24h** | -1.227832028 | 8.20119121 | 1 | -27.37983882 | 24.92417476 |
|  | **Spike 8h** | **Untreated cells 3h** | 81.881503500000* | 8.20119121 | 0 | 55.72949671 | 108.0335103 |
|  |  | **Spike 3h** | 69.832503890000* | 8.20119121 | 0 | 43.6804971 | 95.98451068 |
|  |  | **Untreated cells 8h** | 81.546285700000* | 8.20119121 | 0 | 55.39427891 | 107.6982925 |
|  |  | **Untreated cells 24h** | 81.828950230000* | 8.20119121 | 0 | 55.67694344 | 107.980957 |
|  |  | **Spike 24h** | 80.318453670000* | 8.20119121 | 0 | 54.16644688 | 106.4704605 |
|  | **Untreated cells 24h** | **Untreated cells 3h** | 0.052553267 | 8.20119121 | 1 | -26.09945352 | 26.20456006 |
|  |  | **Spike 3h** | -11.99644634 | 8.20119121 | 1 | -38.14845313 | 14.15556045 |
|  |  | **Untreated cells 8h** | -0.282664529 | 8.20119121 | 1 | -26.43467132 | 25.86934226 |
|  |  | **Spike 8h** | -81.828950230000* | 8.20119121 | 0 | -107.980957 | -55.67694344 |
|  |  | **Spike 24h** | -1.510496558 | 8.20119121 | 1 | -27.66250335 | 24.64151023 |
|  | **Spike 24h** | **Untreated cells 3h** | 1.563049824 | 8.20119121 | 1 | -24.58895697 | 27.71505661 |
|  |  | **Spike 3h** | -10.48594978 | 8.20119121 | 1 | -36.63795657 | 15.66605701 |
|  |  | **Untreated cells 8h** | 1.227832028 | 8.20119121 | 1 | -24.92417476 | 27.37983882 |
|  |  | **Spike 8h** | -80.318453670000* | 8.20119121 | 0 | -106.4704605 | -54.16644688 |
|  |  | **Untreated cells 24h** | 1.510496558 | 8.20119121 | 1 | -24.64151023 | 27.66250335 |
| **IL-17** | **Untreated cells 3h** | **Spike 3h** | -1.509216328 | 3.52472746 | 1 | -12.74888799 | 9.730455336 |
|  |  | **Untreated cells 8h** | -3.263022669 | 3.52472746 | 1 | -14.50269433 | 7.976648995 |
|  |  | **Spike 8h** | -15.993645370000* | 3.52472746 | 0.001 | -27.23331704 | -4.753973709 |
|  |  | **Untreated cells 24h** | -18.056469240000* | 3.52472746 | 0 | -29.2961409 | -6.816797575 |
|  |  | **Spike 24h** | -51.954890770000* | 3.52472746 | 0 | -63.19456243 | -40.7152191 |
|  | **Spike 3h** | **Untreated cells 3h** | 1.509216328 | 3.52472746 | 1 | -9.730455336 | 12.74888799 |
|  |  | **Untreated cells 8h** | -1.753806342 | 3.52472746 | 1 | -12.99347801 | 9.485865322 |
|  |  | **Spike 8h** | -14.484429050000* | 3.52472746 | 0.004 | -25.72410071 | -3.244757381 |
|  |  | **Untreated cells 24h** | -16.547252910000* | 3.52472746 | 0.001 | -27.78692458 | -5.307581248 |
|  |  | **Spike 24h** | -50.445674440000* | 3.52472746 | 0 | -61.6853461 | -39.20600278 |
|  | **Untreated cells 8h** | **Untreated cells 3h** | 3.263022669 | 3.52472746 | 1 | -7.976648995 | 14.50269433 |
|  |  | **Spike 3h** | 1.753806342 | 3.52472746 | 1 | -9.485865322 | 12.99347801 |
|  |  | **Spike 8h** | -12.730622700000* | 3.52472746 | 0.016 | -23.97029437 | -1.490951039 |
|  |  | **Untreated cells 24h** | -14.793446570000* | 3.52472746 | 0.003 | -26.03311823 | -3.553774906 |
|  |  | **Spike 24h** | -48.691868100000* | 3.52472746 | 0 | -59.93153976 | -37.45219643 |
|  | **Spike 8h** | **Untreated cells 3h** | 15.993645370000* | 3.52472746 | 0.001 | 4.753973709 | 27.23331704 |
|  |  | **Spike 3h** | 14.484429050000* | 3.52472746 | 0.004 | 3.244757381 | 25.72410071 |
|  |  | **Untreated cells 8h** | 12.730622700000* | 3.52472746 | 0.016 | 1.490951039 | 23.97029437 |
|  |  | **Untreated cells 24h** | -2.062823867 | 3.52472746 | 1 | -13.30249553 | 9.176847797 |
|  |  | **Spike 24h** | -35.961245400000* | 3.52472746 | 0 | -47.20091706 | -24.72157373 |
|  | **Untreated cells 24h** | **Untreated cells 3h** | 18.056469240000* | 3.52472746 | 0 | 6.816797575 | 29.2961409 |
|  |  | **Spike 3h** | 16.547252910000* | 3.52472746 | 0.001 | 5.307581248 | 27.78692458 |
|  |  | **Untreated cells 8h** | 14.793446570000* | 3.52472746 | 0.003 | 3.553774906 | 26.03311823 |
|  |  | **Spike 8h** | 2.062823867 | 3.52472746 | 1 | -9.176847797 | 13.30249553 |
|  |  | **Spike 24h** | -33.898421530000* | 3.52472746 | 0 | -45.13809319 | -22.65874986 |
|  | **Spike 24h** | **Untreated cells 3h** | 51.954890770000* | 3.52472746 | 0 | 40.7152191 | 63.19456243 |
|  |  | **Spike 3h** | 50.445674440000* | 3.52472746 | 0 | 39.20600278 | 61.6853461 |
|  |  | **Untreated cells 8h** | 48.691868100000* | 3.52472746 | 0 | 37.45219643 | 59.93153976 |
|  |  | **Spike 8h** | 35.961245400000* | 3.52472746 | 0 | 24.72157373 | 47.20091706 |
|  |  | **Untreated cells 24h** | 33.898421530000* | 3.52472746 | 0 | 22.65874986 | 45.13809319 |
| **IL-17RA** | **Untreated cells 3h** | **Spike 3h** | 0.082381388 | 3.33730732 | 1 | -10.55964384 | 10.72440661 |
|  |  | **Untreated cells 8h** | -5.065196629 | 3.33730732 | 1 | -15.70722185 | 5.576828596 |
|  |  | **Spike 8h** | -2.574519284 | 3.33730732 | 1 | -13.21654451 | 8.067505941 |
|  |  | **Untreated cells 24h** | -3.002300656 | 3.33730732 | 1 | -13.64432588 | 7.63972457 |
|  |  | **Spike 24h** | -0.399229634 | 3.33730732 | 1 | -11.04125486 | 10.24279559 |
|  | **Spike 3h** | **Untreated cells 3h** | -0.082381388 | 3.33730732 | 1 | -10.72440661 | 10.55964384 |
|  |  | **Untreated cells 8h** | -5.147578016 | 3.33730732 | 1 | -15.78960324 | 5.494447209 |
|  |  | **Spike 8h** | -2.656900672 | 3.33730732 | 1 | -13.2989259 | 7.985124553 |
|  |  | **Untreated cells 24h** | -3.084682043 | 3.33730732 | 1 | -13.72670727 | 7.557343182 |
|  |  | **Spike 24h** | -0.481611021 | 3.33730732 | 1 | -11.12363625 | 10.1604142 |
|  | **Untreated cells 8h** | **Untreated cells 3h** | 5.065196629 | 3.33730732 | 1 | -5.576828596 | 15.70722185 |
|  |  | **Spike 3h** | 5.147578016 | 3.33730732 | 1 | -5.494447209 | 15.78960324 |
|  |  | **Spike 8h** | 2.490677345 | 3.33730732 | 1 | -8.151347881 | 13.13270257 |
|  |  | **Untreated cells 24h** | 2.062895973 | 3.33730732 | 1 | -8.579129252 | 12.7049212 |
|  |  | **Spike 24h** | 4.665966995 | 3.33730732 | 1 | -5.97605823 | 15.30799222 |
|  | **Spike 8h** | **Untreated cells 3h** | 2.574519284 | 3.33730732 | 1 | -8.067505941 | 13.21654451 |
|  |  | **Spike 3h** | 2.656900672 | 3.33730732 | 1 | -7.985124553 | 13.2989259 |
|  |  | **Untreated cells 8h** | -2.490677345 | 3.33730732 | 1 | -13.13270257 | 8.151347881 |
|  |  | **Untreated cells 24h** | -0.427781371 | 3.33730732 | 1 | -11.0698066 | 10.21424385 |
|  |  | **Spike 24h** | 2.17528965 | 3.33730732 | 1 | -8.466735575 | 12.81731488 |
|  | **Untreated cells 24h** | **Untreated cells 3h** | 3.002300656 | 3.33730732 | 1 | -7.63972457 | 13.64432588 |
|  |  | **Spike 3h** | 3.084682043 | 3.33730732 | 1 | -7.557343182 | 13.72670727 |
|  |  | **Untreated cells 8h** | -2.062895973 | 3.33730732 | 1 | -12.7049212 | 8.579129252 |
|  |  | **Spike 8h** | 0.427781371 | 3.33730732 | 1 | -10.21424385 | 11.0698066 |
|  |  | **Spike 24h** | 2.603071022 | 3.33730732 | 1 | -8.038954204 | 13.24509625 |
|  | **Spike 24h** | **Untreated cells 3h** | 0.399229634 | 3.33730732 | 1 | -10.24279559 | 11.04125486 |
|  |  | **Spike 3h** | 0.481611021 | 3.33730732 | 1 | -10.1604142 | 11.12363625 |
|  |  | **Untreated cells 8h** | -4.665966995 | 3.33730732 | 1 | -15.30799222 | 5.97605823 |
|  |  | **Spike 8h** | -2.17528965 | 3.33730732 | 1 | -12.81731488 | 8.466735575 |
|  |  | **Untreated cells 24h** | -2.603071022 | 3.33730732 | 1 | -13.24509625 | 8.038954204 |
| **TNF-α** | **Untreated cells 3h** | **Spike 3h** | -5.016282497 | 2.24011237 | 0.491 | -12.15956602 | 2.127001023 |
|  |  | **Untreated cells 8h** | 0.447457102 | 2.24011237 | 1 | -6.695826418 | 7.590740622 |
|  |  | **Spike 8h** | -52.584567470000* | 2.24011237 | 0 | -59.72785099 | -45.44128395 |
|  |  | **Untreated cells 24h** | -0.944324937 | 2.24011237 | 1 | -8.087608458 | 6.198958583 |
|  |  | **Spike 24h** | -6.116169095 | 2.24011237 | 0.157 | -13.25945262 | 1.027114426 |
|  | **Spike 3h** | **Untreated cells 3h** | 5.016282497 | 2.24011237 | 0.491 | -2.127001023 | 12.15956602 |
|  |  | **Untreated cells 8h** | 5.463739599 | 2.24011237 | 0.313 | -1.679543921 | 12.60702312 |
|  |  | **Spike 8h** | -47.568284970000* | 2.24011237 | 0 | -54.71156849 | -40.42500145 |
|  |  | **Untreated cells 24h** | 4.07195756 | 2.24011237 | 1 | -3.071325961 | 11.21524108 |
|  |  | **Spike 24h** | -1.099886598 | 2.24011237 | 1 | -8.243170118 | 6.043396923 |
|  | **Untreated cells 8h** | **Untreated cells 3h** | -0.447457102 | 2.24011237 | 1 | -7.590740622 | 6.695826418 |
|  |  | **Spike 3h** | -5.463739599 | 2.24011237 | 0.313 | -12.60702312 | 1.679543921 |
|  |  | **Spike 8h** | -53.032024570000* | 2.24011237 | 0 | -60.17530809 | -45.88874105 |
|  |  | **Untreated cells 24h** | -1.391782039 | 2.24011237 | 1 | -8.53506556 | 5.751501481 |
|  |  | **Spike 24h** | -6.563626197 | 2.24011237 | 0.096 | -13.70690972 | 0.579657324 |
|  | **Spike 8h** | **Untreated cells 3h** | 52.584567470000* | 2.24011237 | 0 | 45.44128395 | 59.72785099 |
|  |  | **Spike 3h** | 47.568284970000* | 2.24011237 | 0 | 40.42500145 | 54.71156849 |
|  |  | **Untreated cells 8h** | 53.032024570000* | 2.24011237 | 0 | 45.88874105 | 60.17530809 |
|  |  | **Untreated cells 24h** | 51.640242530000* | 2.24011237 | 0 | 44.49695901 | 58.78352605 |
|  |  | **Spike 24h** | 46.468398370000* | 2.24011237 | 0 | 39.32511485 | 53.61168189 |
|  | **Untreated cells 24h** | **Untreated cells 3h** | 0.944324937 | 2.24011237 | 1 | -6.198958583 | 8.087608458 |
|  |  | **Spike 3h** | -4.07195756 | 2.24011237 | 1 | -11.21524108 | 3.071325961 |
|  |  | **Untreated cells 8h** | 1.391782039 | 2.24011237 | 1 | -5.751501481 | 8.53506556 |
|  |  | **Spike 8h** | -51.640242530000* | 2.24011237 | 0 | -58.78352605 | -44.49695901 |
|  |  | **Spike 24h** | -5.171844158 | 2.24011237 | 0.42 | -12.31512768 | 1.971439363 |
|  | **Spike 24h** | **Untreated cells 3h** | 6.116169095 | 2.24011237 | 0.157 | -1.027114426 | 13.25945262 |
|  |  | **Spike 3h** | 1.099886598 | 2.24011237 | 1 | -6.043396923 | 8.243170118 |
|  |  | **Untreated cells 8h** | 6.563626197 | 2.24011237 | 0.096 | -0.579657324 | 13.70690972 |
|  |  | **Spike 8h** | -46.468398370000* | 2.24011237 | 0 | -53.61168189 | -39.32511485 |
|  |  | **Untreated cells 24h** | 5.171844158 | 2.24011237 | 0.42 | -1.971439363 | 12.31512768 |
| **MCP-1** | **Untreated cells 3h** | **Spike 3h** | 0.08115393 | 1.89380854 | 1 | -5.957833891 | 6.120141751 |
|  |  | **Untreated cells 8h** | -12.245804110000* | 1.89380854 | 0 | -18.28479194 | -6.206816294 |
|  |  | **Spike 8h** | -6.233361378000* | 1.89380854 | 0.038 | -12.2723492 | -0.194373557 |
|  |  | **Untreated cells 24h** | -2.582328722 | 1.89380854 | 1 | -8.621316543 | 3.456659099 |
|  |  | **Spike 24h** | -12.416990160000* | 1.89380854 | 0 | -18.45597798 | -6.378002339 |
|  | **Spike 3h** | **Untreated cells 3h** | -0.08115393 | 1.89380854 | 1 | -6.120141751 | 5.957833891 |
|  |  | **Untreated cells 8h** | -12.326958050000* | 1.89380854 | 0 | -18.36594587 | -6.287970224 |
|  |  | **Spike 8h** | -6.314515308000* | 1.89380854 | 0.034 | -12.35350313 | -0.275527487 |
|  |  | **Untreated cells 24h** | -2.663482653 | 1.89380854 | 1 | -8.702470474 | 3.375505169 |
|  |  | **Spike 24h** | -12.498144090000* | 1.89380854 | 0 | -18.53713191 | -6.459156269 |
|  | **Untreated cells 8h** | **Untreated cells 3h** | 12.245804110000* | 1.89380854 | 0 | 6.206816294 | 18.28479194 |
|  |  | **Spike 3h** | 12.326958050000* | 1.89380854 | 0 | 6.287970224 | 18.36594587 |
|  |  | **Spike 8h** | 6.012442737 | 1.89380854 | 0.052 | -0.026545084 | 12.05143056 |
|  |  | **Untreated cells 24h** | 9.663475393000* | 1.89380854 | 0 | 3.624487572 | 15.70246321 |
|  |  | **Spike 24h** | -0.171186045 | 1.89380854 | 1 | -6.210173866 | 5.867801776 |
|  | **Spike 8h** | **Untreated cells 3h** | 6.233361378000* | 1.89380854 | 0.038 | 0.194373557 | 12.2723492 |
|  |  | **Spike 3h** | 6.314515308000* | 1.89380854 | 0.034 | 0.275527487 | 12.35350313 |
|  |  | **Untreated cells 8h** | -6.012442737 | 1.89380854 | 0.052 | -12.05143056 | 0.026545084 |
|  |  | **Untreated cells 24h** | 3.651032656 | 1.89380854 | 0.951 | -2.387955165 | 9.690020477 |
|  |  | **Spike 24h** | -6.183628782000* | 1.89380854 | 0.041 | -12.2226166 | -0.144640961 |
|  | **Untreated cells 24h** | **Untreated cells 3h** | 2.582328722 | 1.89380854 | 1 | -3.456659099 | 8.621316543 |
|  |  | **Spike 3h** | 2.663482653 | 1.89380854 | 1 | -3.375505169 | 8.702470474 |
|  |  | **Untreated cells 8h** | -9.663475393000* | 1.89380854 | 0 | -15.70246321 | -3.624487572 |
|  |  | **Spike 8h** | -3.651032656 | 1.89380854 | 0.951 | -9.690020477 | 2.387955165 |
|  |  | **Spike 24h** | -9.834661438000* | 1.89380854 | 0 | -15.87364926 | -3.795673617 |
|  | **Spike 24h** | **Untreated cells 3h** | 12.416990160000* | 1.89380854 | 0 | 6.378002339 | 18.45597798 |
|  |  | **Spike 3h** | 12.498144090000* | 1.89380854 | 0 | 6.459156269 | 18.53713191 |
|  |  | **Untreated cells 8h** | 0.171186045 | 1.89380854 | 1 | -5.867801776 | 6.210173866 |
|  |  | **Spike 8h** | 6.183628782000* | 1.89380854 | 0.041 | 0.144640961 | 12.2226166 |
|  |  | **Untreated cells 24h** | 9.834661438000* | 1.89380854 | 0 | 3.795673617 | 15.87364926 |
| **INF-α** | **Untreated cells 3h** | **Spike 3h** | -4.794503742000* | 1.35410444 | 0.02 | -9.112479953 | -0.47652753 |
|  |  | **Untreated cells 8h** | -2.754858807 | 1.35410444 | 0.762 | -7.072835018 | 1.563117404 |
|  |  | **Spike 8h** | -6.259114962000* | 1.35410444 | 0.001 | -10.57709117 | -1.941138751 |
|  |  | **Untreated cells 24h** | -0.424735486 | 1.35410444 | 1 | -4.742711698 | 3.893240725 |
|  |  | **Spike 24h** | -6.196130470000* | 1.35410444 | 0.001 | -10.51410668 | -1.878154259 |
|  | **Spike 3h** | **Untreated cells 3h** | 4.794503742000* | 1.35410444 | 0.02 | 0.47652753 | 9.112479953 |
|  |  | **Untreated cells 8h** | 2.039644935 | 1.35410444 | 1 | -2.278331276 | 6.357621146 |
|  |  | **Spike 8h** | -1.464611221 | 1.35410444 | 1 | -5.782587432 | 2.853364991 |
|  |  | **Untreated cells 24h** | 4.369768255000* | 1.35410444 | 0.045 | 0.051792044 | 8.687744467 |
|  |  | **Spike 24h** | -1.401626729 | 1.35410444 | 1 | -5.71960294 | 2.916349483 |
|  | **Untreated cells 8h** | **Untreated cells 3h** | 2.754858807 | 1.35410444 | 0.762 | -1.563117404 | 7.072835018 |
|  |  | **Spike 3h** | -2.039644935 | 1.35410444 | 1 | -6.357621146 | 2.278331276 |
|  |  | **Spike 8h** | -3.504256155 | 1.35410444 | 0.221 | -7.822232367 | 0.813720056 |
|  |  | **Untreated cells 24h** | 2.33012332 | 1.35410444 | 1 | -1.987852891 | 6.648099532 |
|  |  | **Spike 24h** | -3.441271664 | 1.35410444 | 0.247 | -7.759247875 | 0.876704548 |
|  | **Spike 8h** | **Untreated cells 3h** | 6.259114962000* | 1.35410444 | 0.001 | 1.941138751 | 10.57709117 |
|  |  | **Spike 3h** | 1.464611221 | 1.35410444 | 1 | -2.853364991 | 5.782587432 |
|  |  | **Untreated cells 8h** | 3.504256155 | 1.35410444 | 0.221 | -0.813720056 | 7.822232367 |
|  |  | **Untreated cells 24h** | 5.834379476000* | 1.35410444 | 0.002 | 1.516403265 | 10.15235569 |
|  |  | **Spike 24h** | 0.062984492 | 1.35410444 | 1 | -4.254991719 | 4.380960703 |
|  | **Untreated cells 24h** | **Untreated cells 3h** | 0.424735486 | 1.35410444 | 1 | -3.893240725 | 4.742711698 |
|  |  | **Spike 3h** | -4.369768255000* | 1.35410444 | 0.045 | -8.687744467 | -0.051792044 |
|  |  | **Untreated cells 8h** | -2.33012332 | 1.35410444 | 1 | -6.648099532 | 1.987852891 |
|  |  | **Spike 8h** | -5.834379476000* | 1.35410444 | 0.002 | -10.15235569 | -1.516403265 |
|  |  | **Spike 24h** | -5.771394984000* | 1.35410444 | 0.003 | -10.0893712 | -1.453418773 |
|  | **Spike 24h** | **Untreated cells 3h** | 6.196130470000* | 1.35410444 | 0.001 | 1.878154259 | 10.51410668 |
|  |  | **Spike 3h** | 1.401626729 | 1.35410444 | 1 | -2.916349483 | 5.71960294 |
|  |  | **Untreated cells 8h** | 3.441271664 | 1.35410444 | 0.247 | -0.876704548 | 7.759247875 |
|  |  | **Spike 8h** | -0.062984492 | 1.35410444 | 1 | -4.380960703 | 4.254991719 |
|  |  | **Untreated cells 24h** | 5.771394984000* | 1.35410444 | 0.003 | 1.453418773 | 10.0893712 |
| **INF-β** | **Untreated cells 3h** | **Spike 3h** | -16.742375640000* | 2.55956894 | 0 | -24.90434415 | -8.580407143 |
|  |  | **Untreated cells 8h** | -2.092691482 | 2.55956894 | 1 | -10.25465998 | 6.06927702 |
|  |  | **Spike 8h** | -9.897437818000* | 2.55956894 | 0.008 | -18.05940632 | -1.735469316 |
|  |  | **Untreated cells 24h** | -1.378288736 | 2.55956894 | 1 | -9.540257238 | 6.783679766 |
|  |  | **Spike 24h** | -16.603915490000* | 2.55956894 | 0 | -24.76588399 | -8.44194699 |
|  | **Spike 3h** | **Untreated cells 3h** | 16.742375640000* | 2.55956894 | 0 | 8.580407143 | 24.90434415 |
|  |  | **Untreated cells 8h** | 14.649684160000* | 2.55956894 | 0 | 6.487715661 | 22.81165267 |
|  |  | **Spike 8h** | 6.844937827 | 2.55956894 | 0.18 | -1.317030675 | 15.00690633 |
|  |  | **Untreated cells 24h** | 15.364086910000* | 2.55956894 | 0 | 7.202118407 | 23.52605541 |
|  |  | **Spike 24h** | 0.138460153 | 2.55956894 | 1 | -8.023508349 | 8.300428655 |
|  | **Untreated cells 8h** | **Untreated cells 3h** | 2.092691482 | 2.55956894 | 1 | -6.06927702 | 10.25465998 |
|  |  | **Spike 3h** | -14.649684160000* | 2.55956894 | 0 | -22.81165267 | -6.487715661 |
|  |  | **Spike 8h** | -7.804746336 | 2.55956894 | 0.071 | -15.96671484 | 0.357222166 |
|  |  | **Untreated cells 24h** | 0.714402746 | 2.55956894 | 1 | -7.447565756 | 8.876371248 |
|  |  | **Spike 24h** | -14.511224010000* | 2.55956894 | 0 | -22.67319251 | -6.349255508 |
|  | **Spike 8h** | **Untreated cells 3h** | 9.897437818000* | 2.55956894 | 0.008 | 1.735469316 | 18.05940632 |
|  |  | **Spike 3h** | -6.844937827 | 2.55956894 | 0.18 | -15.00690633 | 1.317030675 |
|  |  | **Untreated cells 8h** | 7.804746336 | 2.55956894 | 0.071 | -0.357222166 | 15.96671484 |
|  |  | **Untreated cells 24h** | 8.519149082000* | 2.55956894 | 0.035 | 0.35718058 | 16.68111758 |
|  |  | **Spike 24h** | -6.706477674 | 2.55956894 | 0.205 | -14.86844618 | 1.455490828 |
|  | **Untreated cells 24h** | **Untreated cells 3h** | 1.378288736 | 2.55956894 | 1 | -6.783679766 | 9.540257238 |
|  |  | **Spike 3h** | -15.364086910000* | 2.55956894 | 0 | -23.52605541 | -7.202118407 |
|  |  | **Untreated cells 8h** | -0.714402746 | 2.55956894 | 1 | -8.876371248 | 7.447565756 |
|  |  | **Spike 8h** | -8.519149082000* | 2.55956894 | 0.035 | -16.68111758 | -0.35718058 |
|  |  | **Spike 24h** | -15.225626760000* | 2.55956894 | 0 | -23.38759526 | -7.063658254 |
|  | **Spike 24h** | **Untreated cells 3h** | 16.603915490000* | 2.55956894 | 0 | 8.44194699 | 24.76588399 |
|  |  | **Spike 3h** | -0.138460153 | 2.55956894 | 1 | -8.300428655 | 8.023508349 |
|  |  | **Untreated cells 8h** | 14.511224010000* | 2.55956894 | 0 | 6.349255508 | 22.67319251 |
|  |  | **Spike 8h** | 6.706477674 | 2.55956894 | 0.205 | -1.455490828 | 14.86844618 |
|  |  | **Untreated cells 24h** | 15.225626760000* | 2.55956894 | 0 | 7.063658254 | 23.38759526 |
| **INF-γ** | **Untreated cells 3h** | **Spike 3h** | -48.670876620000* | 10.5695729 | 0.001 | -82.37519281 | -14.96656043 |
|  |  | **Untreated cells 8h** | -0.207634901 | 10.5695729 | 1 | -33.91195109 | 33.49668129 |
|  |  | **Spike 8h** | -77.659994610000* | 10.5695729 | 0 | -111.3643108 | -43.95567843 |
|  |  | **Untreated cells 24h** | -0.963183505 | 10.5695729 | 1 | -34.66749969 | 32.74113268 |
|  |  | **Spike 24h** | -10.38193212 | 10.5695729 | 1 | -44.08624831 | 23.32238406 |
|  | **Spike 3h** | **Untreated cells 3h** | 48.670876620000* | 10.5695729 | 0.001 | 14.96656043 | 82.37519281 |
|  |  | **Untreated cells 8h** | 48.463241720000* | 10.5695729 | 0.001 | 14.75892553 | 82.16755791 |
|  |  | **Spike 8h** | -28.98911799 | 10.5695729 | 0.153 | -62.69343418 | 4.715198197 |
|  |  | **Untreated cells 24h** | 47.707693120000* | 10.5695729 | 0.001 | 14.00337693 | 81.41200931 |
|  |  | **Spike 24h** | 38.288944500000* | 10.5695729 | 0.016 | 4.58462831 | 71.99326069 |
|  | **Untreated cells 8h** | **Untreated cells 3h** | 0.207634901 | 10.5695729 | 1 | -33.49668129 | 33.91195109 |
|  |  | **Spike 3h** | -48.463241720000* | 10.5695729 | 0.001 | -82.16755791 | -14.75892553 |
|  |  | **Spike 8h** | -77.452359710000* | 10.5695729 | 0 | -111.1566759 | -43.74804353 |
|  |  | **Untreated cells 24h** | -0.755548604 | 10.5695729 | 1 | -34.45986479 | 32.94876758 |
|  |  | **Spike 24h** | -10.17429722 | 10.5695729 | 1 | -43.87861341 | 23.53001896 |
|  | **Spike 8h** | **Untreated cells 3h** | 77.659994610000* | 10.5695729 | 0 | 43.95567843 | 111.3643108 |
|  |  | **Spike 3h** | 28.98911799 | 10.5695729 | 0.153 | -4.715198197 | 62.69343418 |
|  |  | **Untreated cells 8h** | 77.452359710000* | 10.5695729 | 0 | 43.74804353 | 111.1566759 |
|  |  | **Untreated cells 24h** | 76.696811110000* | 10.5695729 | 0 | 42.99249492 | 110.4011273 |
|  |  | **Spike 24h** | 67.278062490000* | 10.5695729 | 0 | 33.5737463 | 100.9823787 |
|  | **Untreated cells 24h** | **Untreated cells 3h** | 0.963183505 | 10.5695729 | 1 | -32.74113268 | 34.66749969 |
|  |  | **Spike 3h** | -47.707693120000* | 10.5695729 | 0.001 | -81.41200931 | -14.00337693 |
|  |  | **Untreated cells 8h** | 0.755548604 | 10.5695729 | 1 | -32.94876758 | 34.45986479 |
|  |  | **Spike 8h** | -76.696811110000* | 10.5695729 | 0 | -110.4011273 | -42.99249492 |
|  |  | **Spike 24h** | -9.41874862 | 10.5695729 | 1 | -43.12306481 | 24.28556757 |
|  | **Spike 24h** | **Untreated cells 3h** | 10.38193212 | 10.5695729 | 1 | -23.32238406 | 44.08624831 |
|  |  | **Spike 3h** | -38.288944500000* | 10.5695729 | 0.016 | -71.99326069 | -4.58462831 |
|  |  | **Untreated cells 8h** | 10.17429722 | 10.5695729 | 1 | -23.53001896 | 43.87861341 |
|  |  | **Spike 8h** | -67.278062490000* | 10.5695729 | 0 | -100.9823787 | -33.5737463 |
|  |  | **Untreated cells 24h** | 9.41874862 | 10.5695729 | 1 | -24.28556757 | 43.12306481 |
| **TLR-3** | **Untreated cells 3h** | **Spike 3h** | -2.414393361 | 1.00080078 | 0.333 | -5.605752787 | 0.776966064 |
|  |  | **Untreated cells 8h** | -2.658827786 | 1.00080078 | 0.188 | -5.850187212 | 0.532531639 |
|  |  | **Spike 8h** | -10.032778220000* | 1.00080078 | 0 | -13.22413764 | -6.841418793 |
|  |  | **Untreated cells 24h** | -1.390092043 | 1.00080078 | 1 | -4.581451469 | 1.801267383 |
|  |  | **Spike 24h** | -1.176369088 | 1.00080078 | 1 | -4.367728514 | 2.014990338 |
|  | **Spike 3h** | **Untreated cells 3h** | 2.414393361 | 1.00080078 | 0.333 | -0.776966064 | 5.605752787 |
|  |  | **Untreated cells 8h** | -0.244434425 | 1.00080078 | 1 | -3.435793851 | 2.946925001 |
|  |  | **Spike 8h** | -7.618384857000* | 1.00080078 | 0 | -10.80974428 | -4.427025431 |
|  |  | **Untreated cells 24h** | 1.024301318 | 1.00080078 | 1 | -2.167058108 | 4.215660744 |
|  |  | **Spike 24h** | 1.238024274 | 1.00080078 | 1 | -1.953335152 | 4.4293837 |
|  | **Untreated cells 8h** | **Untreated cells 3h** | 2.658827786 | 1.00080078 | 0.188 | -0.532531639 | 5.850187212 |
|  |  | **Spike 3h** | 0.244434425 | 1.00080078 | 1 | -2.946925001 | 3.435793851 |
|  |  | **Spike 8h** | -7.373950432000* | 1.00080078 | 0 | -10.56530986 | -4.182591006 |
|  |  | **Untreated cells 24h** | 1.268735743 | 1.00080078 | 1 | -1.922623683 | 4.460095169 |
|  |  | **Spike 24h** | 1.482458699 | 1.00080078 | 1 | -1.708900727 | 4.673818125 |
|  | **Spike 8h** | **Untreated cells 3h** | 10.032778220000* | 1.00080078 | 0 | 6.841418793 | 13.22413764 |
|  |  | **Spike 3h** | 7.618384857000* | 1.00080078 | 0 | 4.427025431 | 10.80974428 |
|  |  | **Untreated cells 8h** | 7.373950432000* | 1.00080078 | 0 | 4.182591006 | 10.56530986 |
|  |  | **Untreated cells 24h** | 8.642686175000* | 1.00080078 | 0 | 5.451326749 | 11.8340456 |
|  |  | **Spike 24h** | 8.856409131000* | 1.00080078 | 0 | 5.665049705 | 12.04776856 |
|  | **Untreated cells 24h** | **Untreated cells 3h** | 1.390092043 | 1.00080078 | 1 | -1.801267383 | 4.581451469 |
|  |  | **Spike 3h** | -1.024301318 | 1.00080078 | 1 | -4.215660744 | 2.167058108 |
|  |  | **Untreated cells 8h** | -1.268735743 | 1.00080078 | 1 | -4.460095169 | 1.922623683 |
|  |  | **Spike 8h** | -8.642686175000* | 1.00080078 | 0 | -11.8340456 | -5.451326749 |
|  |  | **Spike 24h** | 0.213722955 | 1.00080078 | 1 | -2.97763647 | 3.405082381 |
|  | **Spike 24h** | **Untreated cells 3h** | 1.176369088 | 1.00080078 | 1 | -2.014990338 | 4.367728514 |
|  |  | **Spike 3h** | -1.238024274 | 1.00080078 | 1 | -4.4293837 | 1.953335152 |
|  |  | **Untreated cells 8h** | -1.482458699 | 1.00080078 | 1 | -4.673818125 | 1.708900727 |
|  |  | **Spike 8h** | -8.856409131000* | 1.00080078 | 0 | -12.04776856 | -5.665049705 |
|  |  | **Untreated cells 24h** | -0.213722955 | 1.00080078 | 1 | -3.405082381 | 2.97763647 |
| **TLR-4** | **Untreated cells 3h** | **Spike 3h** | -7.664131784 | 5.67320547 | 1 | -25.75488278 | 10.42661921 |
|  |  | **Untreated cells 8h** | -3.227873048 | 5.67320547 | 1 | -21.31862404 | 14.86287794 |
|  |  | **Spike 8h** | -11.59752054 | 5.67320547 | 0.747 | -29.68827154 | 6.493230447 |
|  |  | **Untreated cells 24h** | -1.892322066 | 5.67320547 | 1 | -19.98307306 | 16.19842893 |
|  |  | **Spike 24h** | -31.297179580000* | 5.67320547 | 0 | -49.38793058 | -13.20642859 |
|  | **Spike 3h** | **Untreated cells 3h** | 7.664131784 | 5.67320547 | 1 | -10.42661921 | 25.75488278 |
|  |  | **Untreated cells 8h** | 4.436258736 | 5.67320547 | 1 | -13.65449226 | 22.52700973 |
|  |  | **Spike 8h** | -3.93338876 | 5.67320547 | 1 | -22.02413975 | 14.15736223 |
|  |  | **Untreated cells 24h** | 5.771809718 | 5.67320547 | 1 | -12.31894127 | 23.86256071 |
|  |  | **Spike 24h** | -23.633047800000* | 5.67320547 | 0.004 | -41.72379879 | -5.542296807 |
|  | **Untreated cells 8h** | **Untreated cells 3h** | 3.227873048 | 5.67320547 | 1 | -14.86287794 | 21.31862404 |
|  |  | **Spike 3h** | -4.436258736 | 5.67320547 | 1 | -22.52700973 | 13.65449226 |
|  |  | **Spike 8h** | -8.369647497 | 5.67320547 | 1 | -26.46039849 | 9.721103495 |
|  |  | **Untreated cells 24h** | 1.335550982 | 5.67320547 | 1 | -16.75520001 | 19.42630197 |
|  |  | **Spike 24h** | -28.069306540000* | 5.67320547 | 0 | -46.16005753 | -9.978555544 |
|  | **Spike 8h** | **Untreated cells 3h** | 11.59752054 | 5.67320547 | 0.747 | -6.493230447 | 29.68827154 |
|  |  | **Spike 3h** | 3.93338876 | 5.67320547 | 1 | -14.15736223 | 22.02413975 |
|  |  | **Untreated cells 8h** | 8.369647497 | 5.67320547 | 1 | -9.721103495 | 26.46039849 |
|  |  | **Untreated cells 24h** | 9.705198479 | 5.67320547 | 1 | -8.385552513 | 27.79594947 |
|  |  | **Spike 24h** | -19.699659040000* | 5.67320547 | 0.024 | -37.79041003 | -1.608908047 |
|  | **Untreated cells 24h** | **Untreated cells 3h** | 1.892322066 | 5.67320547 | 1 | -16.19842893 | 19.98307306 |
|  |  | **Spike 3h** | -5.771809718 | 5.67320547 | 1 | -23.86256071 | 12.31894127 |
|  |  | **Untreated cells 8h** | -1.335550982 | 5.67320547 | 1 | -19.42630197 | 16.75520001 |
|  |  | **Spike 8h** | -9.705198479 | 5.67320547 | 1 | -27.79594947 | 8.385552513 |
|  |  | **Spike 24h** | -29.404857520000* | 5.67320547 | 0 | -47.49560851 | -11.31410653 |
|  | **Spike 24h** | **Untreated cells 3h** | 31.297179580000* | 5.67320547 | 0 | 13.20642859 | 49.38793058 |
|  |  | **Spike 3h** | 23.633047800000* | 5.67320547 | 0.004 | 5.542296807 | 41.72379879 |
|  |  | **Untreated cells 8h** | 28.069306540000* | 5.67320547 | 0 | 9.978555544 | 46.16005753 |
|  |  | **Spike 8h** | 19.699659040000* | 5.67320547 | 0.024 | 1.608908047 | 37.79041003 |
|  |  | **Untreated cells 24h** | 29.404857520000* | 5.67320547 | 0 | 11.31410653 | 47.49560851 |
| **TLR-7** | **Untreated cells 3h** | **Spike 3h** | -13.078318090000* | 3.5552893 | 0.014 | -24.41544552 | -1.741190656 |
|  |  | **Untreated cells 8h** | -10.71598286 | 3.5552893 | 0.078 | -22.05311029 | 0.621144566 |
|  |  | **Spike 8h** | -32.394360140000* | 3.5552893 | 0 | -43.73148757 | -21.05723271 |
|  |  | **Untreated cells 24h** | -4.053611858 | 3.5552893 | 1 | -15.39073929 | 7.283515572 |
|  |  | **Spike 24h** | -15.909164730000* | 3.5552893 | 0.002 | -27.24629216 | -4.572037297 |
|  | **Spike 3h** | **Untreated cells 3h** | 13.078318090000* | 3.5552893 | 0.014 | 1.741190656 | 24.41544552 |
|  |  | **Untreated cells 8h** | 2.362335222 | 3.5552893 | 1 | -8.974792208 | 13.69946265 |
|  |  | **Spike 8h** | -19.316042050000* | 3.5552893 | 0 | -30.65316948 | -7.978914622 |
|  |  | **Untreated cells 24h** | 9.024706229 | 3.5552893 | 0.248 | -2.312421202 | 20.36183366 |
|  |  | **Spike 24h** | -2.830846641 | 3.5552893 | 1 | -14.16797407 | 8.50628079 |
|  | **Untreated cells 8h** | **Untreated cells 3h** | 10.71598286 | 3.5552893 | 0.078 | -0.621144566 | 22.05311029 |
|  |  | **Spike 3h** | -2.362335222 | 3.5552893 | 1 | -13.69946265 | 8.974792208 |
|  |  | **Spike 8h** | -21.678377270000* | 3.5552893 | 0 | -33.0155047 | -10.34124984 |
|  |  | **Untreated cells 24h** | 6.662371006 | 3.5552893 | 1 | -4.674756424 | 17.99949844 |
|  |  | **Spike 24h** | -5.193181863 | 3.5552893 | 1 | -16.53030929 | 6.143945567 |
|  | **Spike 8h** | **Untreated cells 3h** | 32.394360140000* | 3.5552893 | 0 | 21.05723271 | 43.73148757 |
|  |  | **Spike 3h** | 19.316042050000* | 3.5552893 | 0 | 7.978914622 | 30.65316948 |
|  |  | **Untreated cells 8h** | 21.678377270000* | 3.5552893 | 0 | 10.34124984 | 33.0155047 |
|  |  | **Untreated cells 24h** | 28.340748280000* | 3.5552893 | 0 | 17.00362085 | 39.67787571 |
|  |  | **Spike 24h** | 16.485195410000* | 3.5552893 | 0.001 | 5.148067981 | 27.82232284 |
|  | **Untreated cells 24h** | **Untreated cells 3h** | 4.053611858 | 3.5552893 | 1 | -7.283515572 | 15.39073929 |
|  |  | **Spike 3h** | -9.024706229 | 3.5552893 | 0.248 | -20.36183366 | 2.312421202 |
|  |  | **Untreated cells 8h** | -6.662371006 | 3.5552893 | 1 | -17.99949844 | 4.674756424 |
|  |  | **Spike 8h** | -28.340748280000* | 3.5552893 | 0 | -39.67787571 | -17.00362085 |
|  |  | **Spike 24h** | -11.855552870000* | 3.5552893 | 0.034 | -23.1926803 | -0.518425439 |
|  | **Spike 24h** | **Untreated cells 3h** | 15.909164730000* | 3.5552893 | 0.002 | 4.572037297 | 27.24629216 |
|  |  | **Spike 3h** | 2.830846641 | 3.5552893 | 1 | -8.50628079 | 14.16797407 |
|  |  | **Untreated cells 8h** | 5.193181863 | 3.5552893 | 1 | -6.143945567 | 16.53030929 |
|  |  | **Spike 8h** | -16.485195410000* | 3.5552893 | 0.001 | -27.82232284 | -5.148067981 |
|  |  | **Untreated cells 24h** | 11.855552870000* | 3.5552893 | 0.034 | 0.518425439 | 23.1926803 |
| **ACE2** | **Untreated cells 3h** | **Spike 3h** | -5.566338331000* | 0.92285837 | 0 | -8.509154517 | -2.623522146 |
|  |  | **Untreated cells 8h** | -0.93649427 | 0.92285837 | 1 | -3.879310456 | 2.006321915 |
|  |  | **Spike 8h** | -4.445803386000* | 0.92285837 | 0.001 | -7.388619572 | -1.502987201 |
|  |  | **Untreated cells 24h** | -0.005764471 | 0.92285837 | 1 | -2.948580656 | 2.937051715 |
|  |  | **Spike 24h** | -0.147436409 | 0.92285837 | 1 | -3.090252594 | 2.795379777 |
|  | **Spike 3h** | **Untreated cells 3h** | 5.566338331000* | 0.92285837 | 0 | 2.623522146 | 8.509154517 |
|  |  | **Untreated cells 8h** | 4.629844061000* | 0.92285837 | 0 | 1.687027875 | 7.572660246 |
|  |  | **Spike 8h** | 1.120534945 | 0.92285837 | 1 | -1.822281241 | 4.06335113 |
|  |  | **Untreated cells 24h** | 5.560573861000* | 0.92285837 | 0 | 2.617757675 | 8.503390046 |
|  |  | **Spike 24h** | 5.418901922000* | 0.92285837 | 0 | 2.476085737 | 8.361718108 |
|  | **Untreated cells 8h** | **Untreated cells 3h** | 0.93649427 | 0.92285837 | 1 | -2.006321915 | 3.879310456 |
|  |  | **Spike 3h** | -4.629844061000* | 0.92285837 | 0 | -7.572660246 | -1.687027875 |
|  |  | **Spike 8h** | -3.509309116000* | 0.92285837 | 0.01 | -6.452125302 | -0.566492931 |
|  |  | **Untreated cells 24h** | 0.9307298 | 0.92285837 | 1 | -2.012086386 | 3.873545985 |
|  |  | **Spike 24h** | 0.789057861 | 0.92285837 | 1 | -2.153758324 | 3.731874047 |
|  | **Spike 8h** | **Untreated cells 3h** | 4.445803386000* | 0.92285837 | 0.001 | 1.502987201 | 7.388619572 |
|  |  | **Spike 3h** | -1.120534945 | 0.92285837 | 1 | -4.06335113 | 1.822281241 |
|  |  | **Untreated cells 8h** | 3.509309116000* | 0.92285837 | 0.01 | 0.566492931 | 6.452125302 |
|  |  | **Untreated cells 24h** | 4.440038916000* | 0.92285837 | 0.001 | 1.497222731 | 7.382855101 |
|  |  | **Spike 24h** | 4.298366978000* | 0.92285837 | 0.001 | 1.355550792 | 7.241183163 |
|  | **Untreated cells 24h** | **Untreated cells 3h** | 0.005764471 | 0.92285837 | 1 | -2.937051715 | 2.948580656 |
|  |  | **Spike 3h** | -5.560573861000* | 0.92285837 | 0 | -8.503390046 | -2.617757675 |
|  |  | **Untreated cells 8h** | -0.9307298 | 0.92285837 | 1 | -3.873545985 | 2.012086386 |
|  |  | **Spike 8h** | -4.440038916000* | 0.92285837 | 0.001 | -7.382855101 | -1.497222731 |
|  |  | **Spike 24h** | -0.141671938 | 0.92285837 | 1 | -3.084488124 | 2.801144247 |
|  | **Spike 24h** | **Untreated cells 3h** | 0.147436409 | 0.92285837 | 1 | -2.795379777 | 3.090252594 |
|  |  | **Spike 3h** | -5.418901922000* | 0.92285837 | 0 | -8.361718108 | -2.476085737 |
|  |  | **Untreated cells 8h** | -0.789057861 | 0.92285837 | 1 | -3.731874047 | 2.153758324 |
|  |  | **Spike 8h** | -4.298366978000* | 0.92285837 | 0.001 | -7.241183163 | -1.355550792 |
|  |  | **Untreated cells 24h** | 0.141671938 | 0.92285837 | 1 | -2.801144247 | 3.084488124 |
| *** The mean difference is significant at the 0.05 level.** | | |  |  |  |  |  |

**
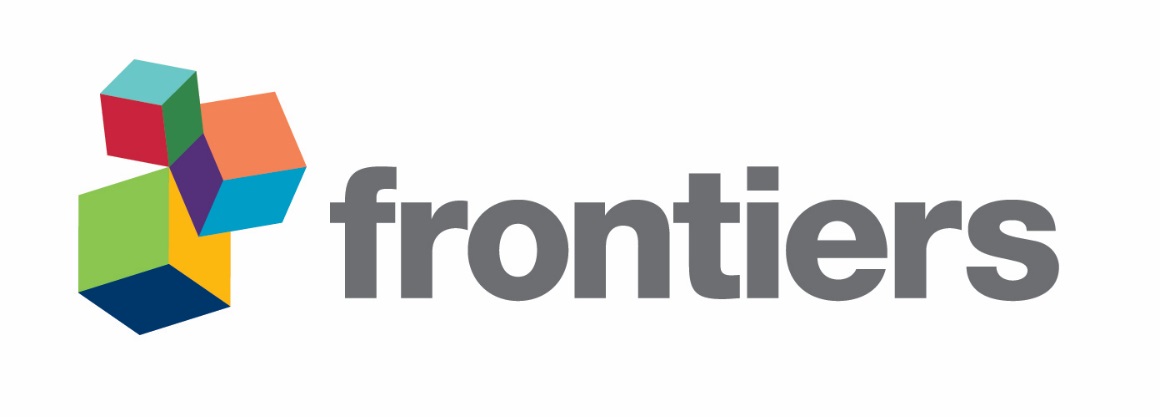
**

.
